# Lethal Canine Distemper Virus (*Morbillivirus canis*) Outbreak in Free-Ranging Black-Tufted Marmosets (*Callithrix penicillata*) in Brazil: Clinical, Pathological, Genotypical Evaluation, and Assessment of Viral Tropism

**DOI:** 10.1155/tbed/4701926

**Published:** 2025-11-12

**Authors:** Bruna Hermine de Campos, Daniel Oliveira dos Santos, Janaina Ribeiro Duarte, Vinicius Henrique Barbosa Amaral, Caio de Castro Cunha Figueiredo, André Duarte Vieira, Letícia Neves Ribeiro, Lucas dos Reis Souza, Nayara Ferreira de Paula, Grazielle Cossenzo Florentino Galinari, Sara Cândida Ferreira dos Santos, Talita Emile Ribeiro Adelino, Paulo Eduardo de Souza da Silva, Natália Rocha Guimarães, Luiz Marcelo Ribeiro Tomé, Maurício Teixeira Lima, Carlyle Mendes Coelho, Herlandes Penha Tinoco, Peter MacGarr Rabinowitz, Luiz Carlos Junior Alcantara, Marta Giovanetti, Felipe Campos de Melo Iani, Erica Azevedo Costa, Ayisa Rodrigues de Oliveira, Renato Lima Santos, Marcelo Pires Nogueira de Carvalho

**Affiliations:** ^1^Department of Veterinary Clinical and Surgical Sciences, School of Veterinary Medicine, Federal University of Minas Gerais, Belo Horizonte, Minas Gerais, Brazil; ^2^Department of Preventive Veterinary Medicine, School of Veterinary Medicine, Federal University of Minas Gerais, Belo Horizonte, Minas Gerais, Brazil; ^3^Virology and Rickettsioses Service, Ezequiel Dias Foundation, Belo Horizonte, Minas Gerais, Brazil; ^4^Department of Microbiology, Federal University of Minas Gerais, Belo Horizonte, Minas Gerais, Brazil; ^5^Veterinary Management Division, Foundation of Municipal Parks and Zoobotany of Belo Horizonte, Belo Horizonte, Minas Gerais, Brazil; ^6^Department of Environmental and Occupational Health Sciences, University of Washington, Seattle, Washington, USA; ^7^René Rachou Institute, Oswaldo Cruz Foundation, Belo Horizonte, Minas Gerais, Brazil; ^8^Department of Sciences and Technologies for Sustainable Development and One Health, Università Campus Bio-Medico di Roma, Roma, Lazio, Italy

**Keywords:** immunohistochemistry, morbillivirus, primates, public health, urban parks, wildlife

## Abstract

Canine distemper virus (CDV; *Morbilivirus canis*) is a morbillivirus with worldwide distribution. The virus belongs to the family Paramyxoviridae, which includes highly pathogenic viruses such as measles virus (MeV) (*Measles virus*) and rinderpest virus (*Rinderpest morbillivirus*). Canine distemper is primarily a disease in domestic dogs, but it has been described in terrestrial and aquatic wild mammals, demonstrating the ability of CDV to cross species barriers. Here, we describe a lethal CDV outbreak in free-ranging marmosets (*Callithrix penicillata*) in Brazil. The marmosets were captured during a targeted epidemiological surveillance program aimed at monitoring viral agents in wildlife in urban parks of Belo Horizonte, Minas Gerais, Brazil. Affected marmosets presented with neurological signs, and others were found dead in the same area. In this report, we detail the occurrence of the outbreak and describe clinical manifestations, gross and histopathological lesions, tissue distribution of the virus by immunohistochemistry (IHC) and molecular methods, and phylogenetic analyses of the virus. This study demonstrated that CDV can infect free-ranging black-tufted marmosets and lead to neurologic signs, cutaneous lesions, and death. Viral genomic sequences and antigens were detected in several organs, indicating a pantropic distribution of CDV in this neotropical primate species. Additionally, the marmosets were also tested for MeV and *Lyssavirus*, which yielded negative results. Coinfections with CDV and *Callitrichinae gammaherpesvirus* 3 were detected in three marmosets. The CDV sequences identified here were closely related to both South American sequences and Vero cell-adapted lineages. These findings have significant implications from a One Health perspective.

## 1. Introduction

Morbilliviruses significantly impact both human and animal health, causing diseases that are associated with high morbidity and mortality [1]. Canine distemper virus (CDV; *Morbilivirus canis*) remains one of the most fatal infectious diseases in domestic dogs, terrestrial, and aquatic wild mammals [2]. Evidence from natural and experimental infections demonstrated that morbilliviruses can readily cross species barriers [3]. Particularly alarming are reports of natural CDV infection in Old World primates from Japan and China with adapted CDV strains [4–6].

CDV is an enveloped, single-stranded, negative-sense RNA virus that contains six structural proteins and two accessory nonstructural proteins [7]. The emergence of the disease in new host species is associated with mutations affecting the binding site of the H protein to viral entry receptors, such as the signaling lymphocyte activation molecule (SLAM, CD150) and nectin-4 [8–11]. This adaptation is related to the varying rates of morbidity and mortality [3].

Similar to the measles virus (MeV), CDV initially targets lymphoid tissues, causing intense lymphopenia and immunosuppression [12, 13]. Generally, CDV exhibits lympho-, neuro-, and epitheliotropism, resulting in a systemic infection of nearly all organ systems [12, 14]. Infection of the central nervous system (CNS) is common in CDV, whereas this is a rare complication in measles. This difference, along with severe immunosuppression, may explain the high fatality rates observed in canine distemper compared to the low mortality rates reported for measles [15].

Brazil is the seventh most populous country in the world and harbors one of the greatest diversities of primate species, with areas of high priority for primate conservation [16]. New World primates (NWP) are particularly vulnerable to the introduction of exotic and human pathogens, which is facilitated by frequent interspecies interactions [16, 17].

Pathogen transmission from domestic animals to wildlife, not only threatens biodiversity but also poses a significant zoonotic risk [2, 3]. CDV adaptation to primates supports the hypothesis that this virus might also be able to infect humans [4, 5, 6]. Under a One Health perspective, this scenario presents a relevant concern with potential epidemic implications.

In this study, we describe a lethal outbreak caused by CDV in wild black-tufted marmosets (*Callithrix penicillata*) in an urban area of Brazil and focus on the clinical and pathological aspects of the infection and tissue distribution of antigens.

## 2. Materials and Methods

### 2.1. Ethics Approval

All procedures were approved by the Ethics Committee on the Use of Animals of the Universidade Federal de Minas Gerais (CEUA/UFMG) under protocol number 78/2022, as well as by the Instituto Chico Mendes de Conservação de Biodiversidade under protocol number 81392, by the Fundação de Parques Municipais e Zoobotânica de Belo Horizonte (FPMZ-BH) under protocol number FU002/2022, and by Sisgen under protocol AA9C23B.

### 2.2. Description of Cases

During a 39-day period in 2022, seven free-ranging adult black-tufted marmosets (four males and three females) were found dead or captured in a forested urban area at the Belo Horizonte Zoo, which is located in a vast forested area within the urban area of Belo Horizonte (State of Minas Gerais, Brazil). Three marmosets exhibited lethargy before death and two showed severe neurological signs, including generalized myoclonus (Supporting Information 1: Video S1). Euthanasia of the two marmosets with neurological signs was performed by induction with 100% isoflurane inhalation until complete sedation, followed by intravenous administration of propofol (0.8–1 mg/kg) and potassium chloride (35 mg/kg), with continuous cardiorespiratory monitoring until cessation. Oropharyngeal and rectal swabs from live marmosets were sampled and placed in buffered saline solution with 1% antibiotics (200 U/mL of penicillin and 200 μg/mL of streptomycin) and stored at −80°C. Necropsies were performed on all seven marmosets, and tissue samples were fixed in 10% neutral buffered formalin, processed until paraffin embedding, and sectioned for histopathology and immunohistochemistry (IHC). Additional tissue samples were stored at −20°C for molecular analysis.

### 2.3. DNA and RNA Extraction

Extraction and purification of DNA and RNA from the swabs and tissue samples were performed using magnetic beads with the commercial Loccus Extracta Kit Fast—DNA and RNA Viral (MVXA-PV96-B FAST) (Cotia, São Paulo, Brazil) following the manufacturers recommendations. Tissue samples were macerated using the FastPrep-24 5G (MPBio) instrument.

### 2.4. Pan-Herpesvirus Polymerase Chain (PCR)

Total DNA was subjected to herpesvirus detection using a nested consensus pan-herpesvirus PCR targeting a fragment of the DNA polymerase gene [18]. In the first PCR round, three degenerate and dI (2′-deoxyinosine)-substituted primers were applied: 285s DFA (5′-AYTTYGCIAGYYTITAYCC-3′), 285s ILK (5′- TCCTGGACAAGCAGCARIYSGCIMTIAA-3′), and 285as KG1 (5′-GTCTTGCTCACCAGITCIACICCYTT-3′). Reactions underwent 40 cycles: 30 s denaturation at 94°C, 1 min annealing at 46°C, and 1 min chain extension at 72°C in a Veriti 96-well Thermal Cycler. Postcycling, mixtures were incubated for 7 min at 72°C and then held at 4°C.

For the second round, the primers 286sTGV (5′-TGTAACTCGGTGTAYGGITTYACIGGIGT-3′) and 286-as IYG (5′-CACAGAGTCCGTRTCICCRTAIAT-3′) were used. Reactions underwent 40 cycles: 30 s denaturation at 94°C, 1 min annealing at 46°C, and 1 min chain extension at 72°C in a Veriti 96-well Thermal Cycler. Postcycling, mixtures were incubated for 7 min at 72°C and then held at 4°C.

The PCR amplicons were analyzed by 1% (w/v) agarose gel electrophoresis containing 0.5 mg of ethidium bromide per mL, and DNA products were visualized by transillumination with a long-wave UV light box.

For herpesvirus testing, an equid alphaherpesvirus 1 (EHV-1) positive control was used. The positive control consisted of an isolate of a neuropathogenic variant of the virus, previously characterized by Catizane et al. [19]. Negative controls for DNA extraction and PCR reactions (nuclease-free water) were included in all assays to exclude possible contamination. All tests were performed in duplicate.

### 2.5. Measles Real-Time RT-PCR

Total RNA was subjected to molecular diagnosis for MeV using a real-time RT-PCR assay. The test was performed with the GoTaq Probe 1-Step RT-qPCR System (Promega) on the 7500 Real-Time PCR System (Applied Biosystems) following the manufacturer's protocol. Amplification of the viral nucleoprotein (*N*) gene was achieved using a specific set of primers (1139–1160F: TGGCATCTGAACTCGGTATCAC and 1213–1190: TGTCCTCAGTAGTATGCATTGCAA) and a probe (1163–1187P: FAM-CCGAGGATGCAAGGCTTGTTCGA-BHQ1), as previously described [20]. The reaction mixture (25 µL total volume) included 1× GoTaq Probe qPCR Master Mix with dUTP and CXR Reference Dye, 0.3 µM of each primer, 0.1 µM of the probe, 0.5 µL of GoScript RT Mix for 1-Step RT-qPCR, and 5 µL of extracted total RNA. The thermocycling conditions were as follows: reverse transcription at 45°C for 15 min, initial denaturation at 95°C for 2 min, followed by 40 cycles of denaturation at 95°C for 15 s and annealing/extension at 60°C for 60 s.

For MeV testing, the positive control consisted of RNA extracted from an aliquot of the measles-mumps-rubella vaccine (Bio-manguinhos). Negative controls included: extraction negative control (ENC), consisting of an aliquot of HeLa cells processed from the extraction step onward; and reaction negative control (RNC), consisting of all PCR reagents with nuclease-free water replacing the sample at the same volume.

### 2.6. Pan-*Lyssavirus* Real-Time RT-PCR

Total RNA was extracted from the samples and subjected to molecular diagnosis for lyssavirus using a real-time RT-PCR assay. The test was performed with the SuperScript III Platinum One-Step qRT-PCR Kit w/ROX (Thermo Fisher Scientific, USA) on the QuantStudio 5 Real-Time PCR System (Thermo Fisher Scientific), following the manufacturer's instructions and the methodology previously described by Wadhwa et al. [21], with minor adaptations. Amplification targeted a highly conserved region comprising the noncoding leader sequence and part of the nucleoprotein (N) coding sequence of the *lyssavirus* genome.

The assay employed a set of degenerate primers: two forward primers targeting the 5′ end of the rabies virus genome (5′-ACGCTTAACAACCAGATCAAAGAA-3′ and 5′-ACGCTTAACAACAAAATCADDAGAAG-3′) and one reverse primer targeting the *N* gene with six degenerate nucleotides (5′-CMGGGTATTRTAYTCATAYTGRTC-3′). The probe (LN34m) was modified with a minor groove binder (MGB) and a nonfluorescent quencher (NFQ) and labeled with a 5′ 6-FAM fluorescent dye.

Reactions were prepared in a total volume of 12.5 µL, containing 1× reaction mix, 0.6 µM of each primer, 0.2 µM of the probe, and 2.5 µL of extracted RNA, as per the manufacturer's instructions. Thermocycling conditions consisted of reverse transcription at 50°C for 30 min, initial denaturation at 95°C for 2 min, followed by 45 cycles of denaturation at 95°C for 15 s and annealing/extension at 56°C for 30 s. Amplification data were analyzed using the QuantStudio 5 Software (Thermo Fisher Scientific).

A positive control consisting of a known lyssavirus-positive equine CNS sample (provided by Prof. Erica Azevedo Costa, School of Veterinary Medicine, UFMG) was included. Negative controls for RNA extraction and PCR reactions (nuclease-free water) were used in all runs to exclude possible contamination. All assays were performed in duplicate.

### 2.7. CDV RT-PCR

To identify genetic lineages and characterize circulating CDV strains, primers targeting a short region of the fusion (*F*) gene encoding the F protein signal peptide (Fsp) were used. A 681 bp fragment was amplified via RT-PCR using forward (5′-TCCAGGACATAGCAAGCCAACA-3′) and reverse (5′-GGTTGATTGGTTCGAGGACTGAA-3′) primers with the SuperScript III One-Step RT-PCR System and Platinum Taq DNA Polymerase (Invitrogen, Thermo Fisher Scientific, Massachusetts, EUA), following the protocol described by Sarute et al. [22].

Initially, the reverse transcription mixture was held at 50°C for 30 min, followed by 2 min at 94°C for Taq inhibitor activation. Reactions underwent 45 cycles: 30 s denaturation at 94°C, 45 s annealing at 58°C, and 1 min chain extension at 68°C in a Veriti 96-well Thermal Cycler. Postcycling, mixtures were incubated for 7 min at 68°C and then held at 4°C. The RT-PCR amplicons were analyzed by 1% (w/v) agarose gel electrophoresis containing 0.5 mg of ethidium bromide per ml, and DNA products were visualized by transillumination with a long-wave UV light box.

For CDV testing, a positive control provided by Prof. Zélia Inês Portela Lobato (School of Veterinary Medicine, UFMG) was used. Negative controls for RNA extraction and PCR reactions (nuclease-free water) were included in all assays to exclude possible contamination. All tests were performed in duplicate.

### 2.8. Sequencing of the Fusion Protein (*F*) Gene

The amplicons were purified using 1x AMPure XP magnetic beads (Beckman Coulter) and quantified with a Qubit Fluorometric Quantitation 3 (Thermo Fisher Scientific) using the Qubit dsDNA HS Assay Kit (Thermo Fisher Scientific, Massachusetts, EUA). The genomic library was then prepared with the NEBNext Fast DNA Library Prep Set for Ion Torrent (New England Biolabs) and sequenced on the Ion 314 chip using the Ion Torrent PGM platform (Thermo Fisher Scientific), following the manufacturer's instructions. The amplicons were assembled using Geneious Prime 2024.0.3 (https://www.geneious.com) and Minimap2 Version 2.2.0 (*⁣*^*∗*^).

A phylogenetic analysis was conducted using the fusion protein (*F*) gene as a reference. This study encompassed 45 nucleotide sequences, totaling 432 positions. Sequence alignment was performed using MAFFT v1.4.0 in Geneious Prime 2024.0.3 (https://www.geneious.com) [23], which was also used to calculate sequence identity. The phylogenetic tree was reconstructed using IQ-TREE 2.3.6 [24] with 1000 bootstrap replicates, employing the Kimura's three-parameter model with empirical base frequencies and four gamma-distributed rate variations across sites, and edited using iTOL Version 6.9.1 [25]. Likelihood mapping analysis was performed to assess the phylogenetic signal in the dataset, confirming that both the dataset and the analyzed region exhibited a strong phylogenetic signal. Maximum likelihood phylogenetic analysis was performed on 432 base pair nucleotide sequences from the fusion (*F*) gene encoding the Fsp of CDV strains, with node support evaluated using 1000 bootstrap replicates.

### 2.9. CDV (CDV) and Herpesvirus IHC

IHC was performed with a monoclonal anti-CDV antibody (DV2-12 clone, Santa Cruz Biotechnology) and monoclonal antihuman herpesvirus 1 (HVV-1) antibody (20.7.1 clone, Santa Cruz Biotechnology). Antigen retrieval was performed in a pressure cooker with a high pH solution (Dako) for 10 min. Peroxidase blocking was performed with 3.5% hydrogen peroxide solution for 40 min and protein blocking with 6% skim milk solution for 1 h. Primary antibodies were incubated overnight at 4°C, both at 1:100 dilution. EnVision Flex (Dako) secondary antibody was used to develop the reaction and magenta chromogen (Dako) for revealing. Previously tested samples from CDV-positive and *Human alphaherpesvirus 1* (HSV-1)-positive animals were used as positive controls. For negative controls, the primary antibody was substituted by buffer (Supporting Information 2: Figure S1).

## 3. Results

**Figure 1:**
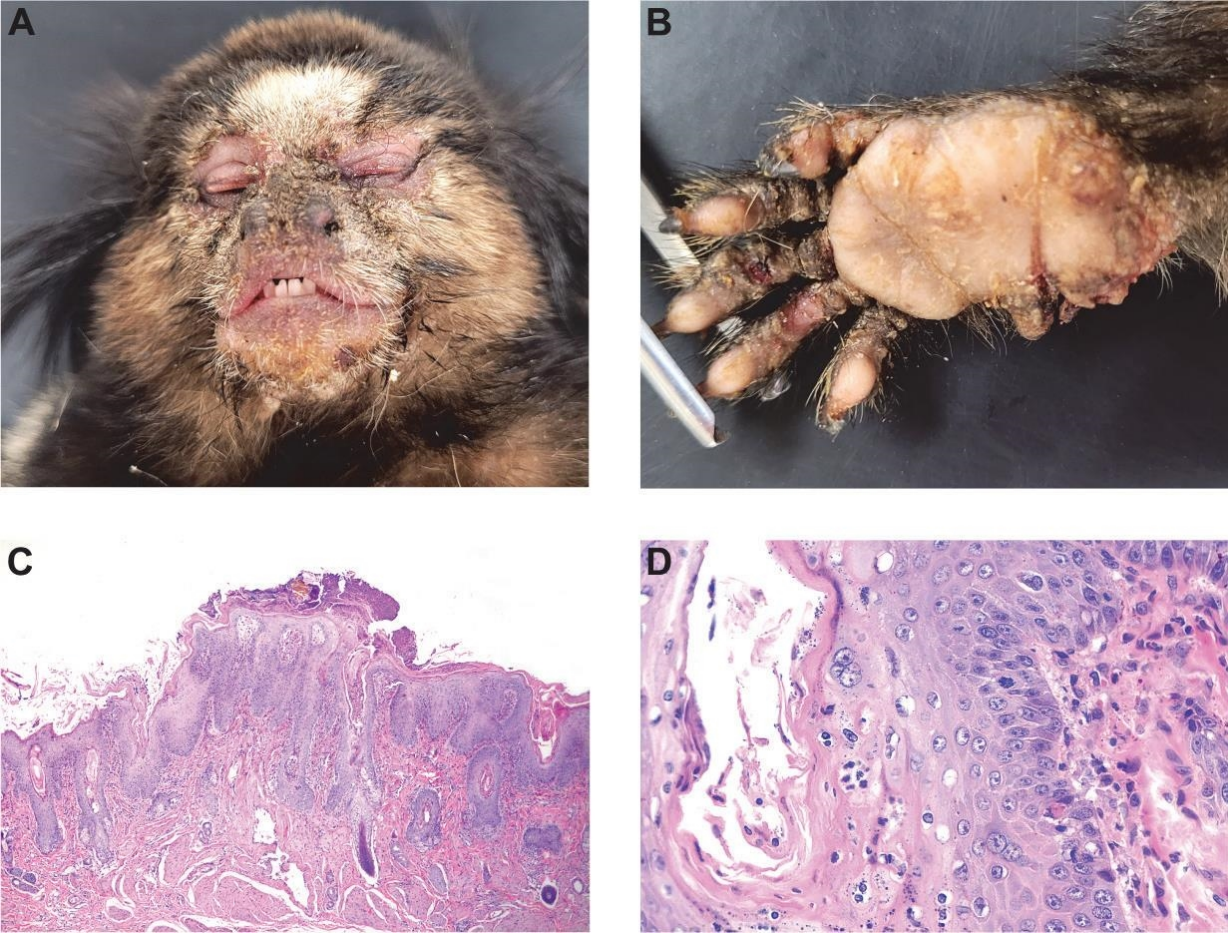
Gross and histological cutaneous lesions of free-ranging black-tufted marmosets (*Callithrix penicillata*) naturally infected with the canine distemper virus. (A) Facial skin with multifocal moderate hyperkeratosis. (B) Right hand with multifocal areas of thickening and hyperkeratosis. (C) Facial skin with diffuse severe acanthosis and moderate hyperkeratosis. HE, 40x. (D) Skin with acanthosis and syncytial epithelial cells. HE, 400x.

**Figure 2:**
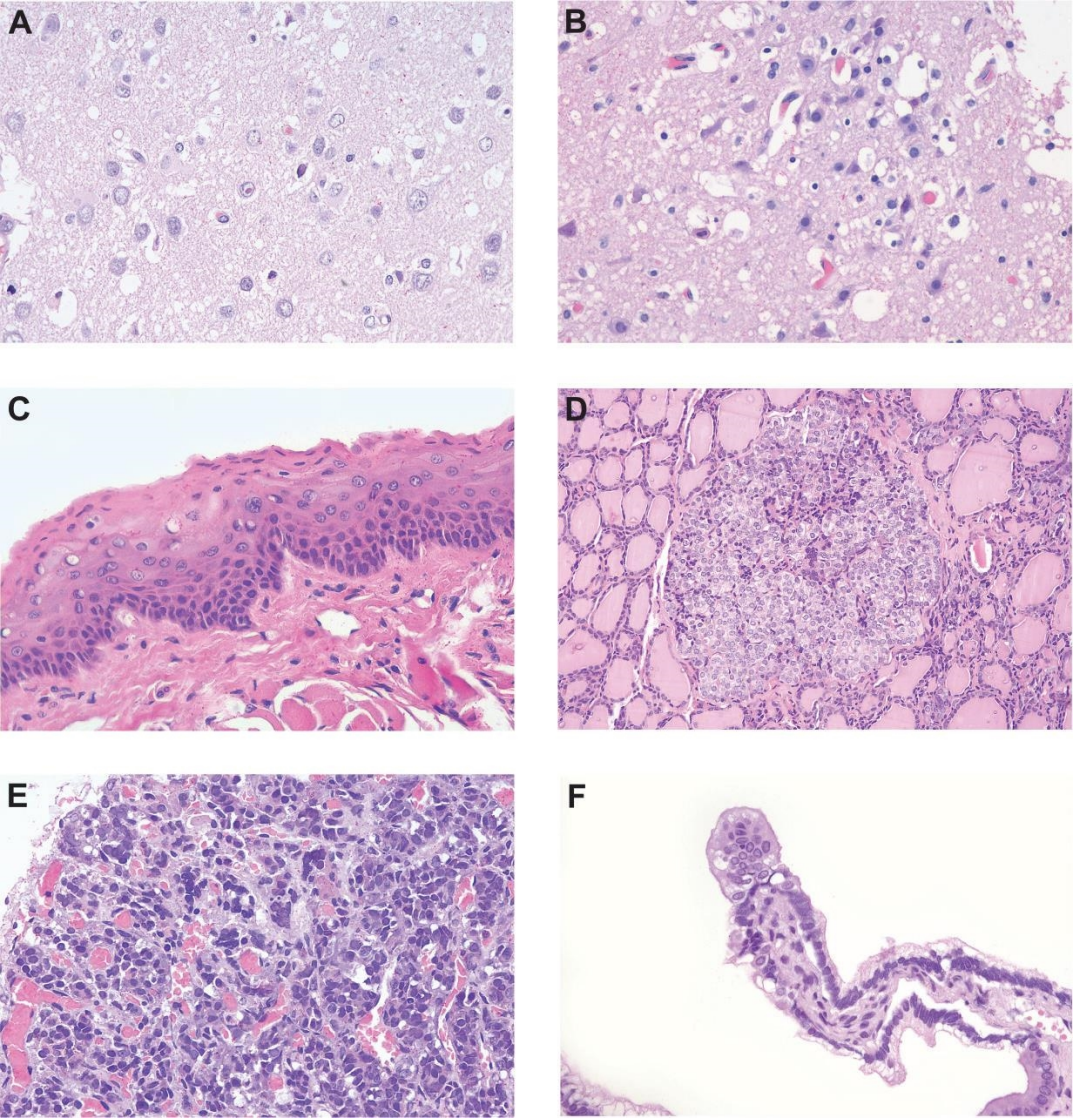
Brain, tongue, parathyroid gland, pituitary gland, and gall bladder of free-ranging black-tufted marmosets (*Callithrix penicillata*) naturally infected with the canine distemper virus. (A) Brain with gliosis and intranuclear eosinophilic inclusion body. HE, 400x. (B) Brain with gliosis and individual neuronal necrosis. HE, 400x. (C) Tongue with epithelial syncytial cells. HE, 400x. (D) Parathyroid gland with syncytial cells. HE, 200x. (E) Pituitary gland with syncytial cells. HE, 400x. (F) Gallbladder with syncytial cells. HE, 400x.

Pathologic findings Grossly, four marmosets had significant skin thickening with crusts on the face, limbs, and inguinal area (Figure 1A,B). Histological lesions are described in Table 1. Histologically, skin lesions consisted of varying degrees of acanthosis (4/7), hyperkeratosis (7/7), follicular keratosis (3/7), epithelial degeneration (4/7), syncytial epithelial cells (5/7), and mild neutrophilic dermatitis (4/7) (Figure 1C,D). Brain lesions included gliosis (5/6) with gemistocytes (2/6) and Alzheimer type II cells (4/6), neuronal necrosis (3/6), meningitis (3/6), choroiditis (1/6), and intranuclear eosinophilic inclusion bodies in neurons and astrocytes (2/6) (Figure 2A,B). Syncytial cells were also observed in the epithelium of the tongue (6/7) (Figure 2C), parathyroid gland (6/6) (Figure 2D), and pituitary gland (1/5) (Figure 2E), gallbladder (1/7) (Figure 2F), urinary bladder (1/5) and esophagus (1/4).

### 3.1. Molecular Investigation and Sequencing of the Fusion Protein (*F*) Gene

**Figure 3:**
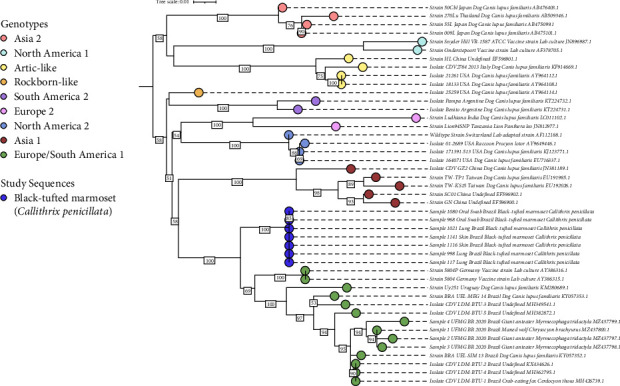
The phylogenetic tree was reconstructed using IQ-TREE 2.3.6 with 1000 bootstrap replicates, employing the Kimura's 3-parameter model with empirical base frequencies and 4 gamma-distributed rate variations across sites, and edited using iTOL Version 6.9.1. Maximum likelihood phylogenetic analysis was performed on 432 base pair nucleotide sequences from the fusion (*F*) gene encoding the signal peptide of the F protein (Fsp) of canine distemper virus strains. Samples labeled in blue indicate those sequenced in this study. Sequences from the literature include their accession numbers at the end. Only bootstrap values ≥50% are shown.

DNA and RNA samples were extracted from oral and rectal swabs, as well as from fresh frozen samples of brain, skin, spleen, liver, kidneys, and lungs for PCR detection of herpesviruses, MeV, *Lyssavirus*, and CDV (Table 2). All samples, with the exception of one rectal swab and kidneys from one marmoset, were positive for CDV (Table 2). A phylogenetic analysis was performed based on the alignment of 45 sequences of a 432 base pair fragment of the fusion (*F*) gene, which encodes the signal peptide of the CDV F protein (Figure 3). Sequence alignment revealed a mean pairwise identity of 99.1% among the marmoset outbreak sequences. The phylogeny showed that the sequences amplified from the seven marmosets clustered together within a clade supported by 100% bootstrap and were most closely related to CDV strains from the Europe/South American 1 genotype, which includes 12 clinical sequences from Brazil and two sequences belonging to the vaccine lineage 5804. Among the Brazilian wild type isolates, the highest similarity was observed with UEL-MEG_14_B, obtained from a domestic dog (GenBank number KY057353.1), with 93.9% identity. When considering only wild hosts, the most similar sequence was an isolate from a crab-eating fox (*Cerdocyon thous*; GenBank number MH426739.1), with 93.06% identity. In comparison, the vaccine sequences 5804 and 5804P (*AY386315.1* and *AY386316.1*, respectively) showed 95.6% pairwise identity.

Brain and skin samples from three marmosets tested positive for a herpesvirus using a pan-herpesvirus PCR. A brain sample from marmoset 1 was sequenced, yielding a 425 bp fragment corresponding to the herpesvirus DNA polymerase. The sequence was identified as *Callitrichine gammaherpesvirus* 3 (CalHV-3), showing 97.65% identity (100% coverage for the amplicon) in the BLAST analysis against the reference sequence (GenBank number NC_004367.1). The obtained sequence has been deposited under number CalHV-3_01_BR PQ362650 (Table 2). Sequencing of the other two pan-herpesvirus-positive marmosets resulted in few reads and an unreliable sequence. All samples were negative for MeV and *Lyssavirus* (Table 2). None of the negative controls yielded any amplicon.

### 3.2. Anti-CDV and Anti-Herpesvirus IHC

IHC was used to evaluate the presence and distribution of CDV and herpesvirus antigens. Samples of brain, skin, tongue, liver, gallbladder, lungs, lymph nodes, spleen, bone marrow, and adrenal gland were evaluated for all marmosets. Ovaries, uterus, and vagina were evaluated for all three females. Other tissues such as esophagus, stomach, small and large intestines, trachea, kidneys, urinary bladder, testes, prostate gland, and thyroid gland were not available from all marmosets due to lack of sampling or autolysis.

There were anti-CDV immunopositive cells in all organs tested by IHC (Table 3). Herpesvirus antigens were detected in brain (3/7), skin (5/7), and tongue (2/7). In contrast, only three marmosets (1, 3, and 7) had a few herpesvirus-positive cells by IHC, including a few neurons in the brain and epidermal and mucosal epithelial cells in the skin and tongue, respectively (Supporting Information 3: Figure S2).

### 3.3. CDV Antigen Tissue Distribution

**Figure 4:**
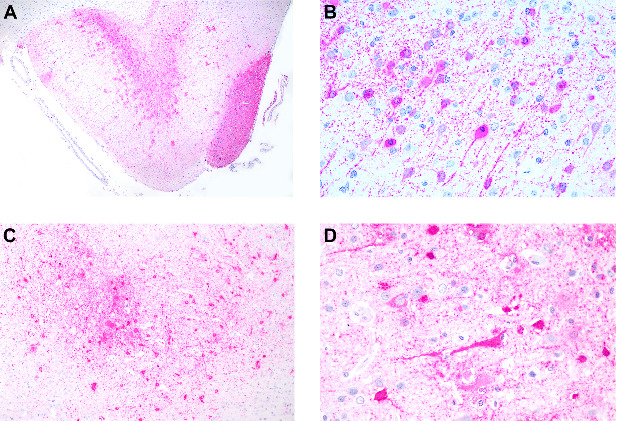
Anticanine distemper virus immunohistochemistry of brains from free-ranging black-tufted marmosets (*Callithrix penicillata*) naturally infected with the canine distemper virus. (A) Brain, frontal lobe with an extensive area of immunostaining. Magenta chromogen, 40x. (B) Brain, frontal lobe with immunostained neurons and glial cells. Magenta chromogen, 400x. (C) Brain, telencephalic cortex with extensive area of immunostaining. Magenta chromogen, 100x. (D) Brain, telencephalic cortex with immunostained neurons and glial cells. Magenta chromogen, 400x.

To further characterize CDV distribution we evaluated the location and intensity of anti-CDV immunostained cells in all organs evaluated as described in the following tables.  Table 4 lists the CDV antigen distribution in brain samples and positive cell types. Various brain segments were evaluated. These included the telencephalic cortex, frontal lobe, occipital lobe, cerebellum, and brain stem in six of the seven CDV-positive marmosets. In the case of marmoset seven, it was impossible to perform a proper evaluation of the brain due to a head trauma with severe brain lacerations. CDV immunostaining was most intense in the frontal lobe (Figure 4A,B) and telencephalic cortex (Figure 4C,D). However, immunolabeling of CDV antigens was also present in the occipital lobe, cerebellum, and brain stem. Antigens were detected in neurons, glial cells, meningeal cells, and choroid plexus cells.

**Figure 5:**
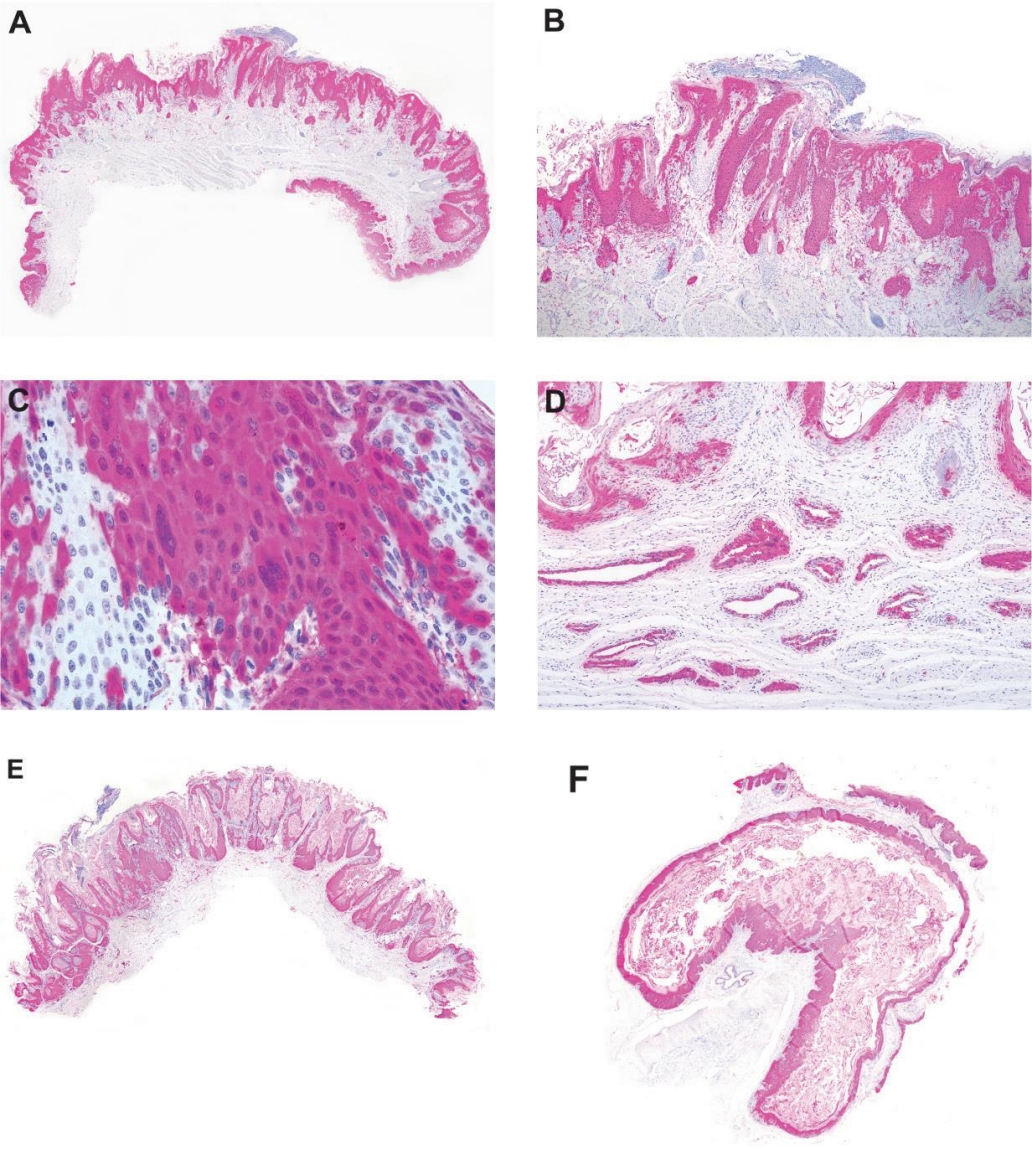
Anticanine distemper virus immunohistochemistry of skin from free-ranging black-tufted marmosets (*Callithrix penicillata*) naturally infected with the canine distemper virus. (A) Facial skin with diffuse immunostaining of the epidermis. Magenta chromogen, subgross view. (B) Facial skin, epidermis with diffuse immunostained epithelial cells. Magenta chromogen, 40x. (C) Facial skin, epidermis with multifocal to coalescent immunostained epithelial cells, including multinucleated syncytial cells. Magenta chromogen, 400x. (D) Facial skin, epidermis, and apocrine glands with multifocal immunostained epithelial cells. Magenta chromogen, 100x. (E) Scrotum, epidermis with diffuse immunostained epithelial cells. Magenta chromogen, subgross view. (F) Penis, penile mucosa, and prepuce epithelium with diffuse immunostained epithelial cells. Magenta chromogen, subgross view.

Skin samples from all marmosets were positive for CDV antigens (Table 5). Facial skin was evaluated in 6/7 marmosets and CDV antigens were found in the epithelial cells of the epidermis, sebaceous glands, and the connective tissue of the superficial dermis (Figure 5A–C). Apocrine glands (Figure 5D) were positive in 4/5 marmosets (two marmosets had no apocrine glands in the evaluated skin samples). In the case of two marmosets, skin samples from the thoracic limb (marmoset 2), perianal (marmosets 2 and 7) and perivulvar (marmoset 2) regions, and ear lobe (marmoset 7) were also evaluated and had similar distributions of antigens. The scrotum was evaluated in all four male marmosets and in all of them epidermis, sebaceous and apocrine glands, and connective tissue of the superficial epidermis were positive (4/4) (Figure 5E). The penis of marmoset 5 had diffuse CDV immunostaining in the mucosal epithelium of the prepuce and penis (Figure 5F).

**Figure 6:**
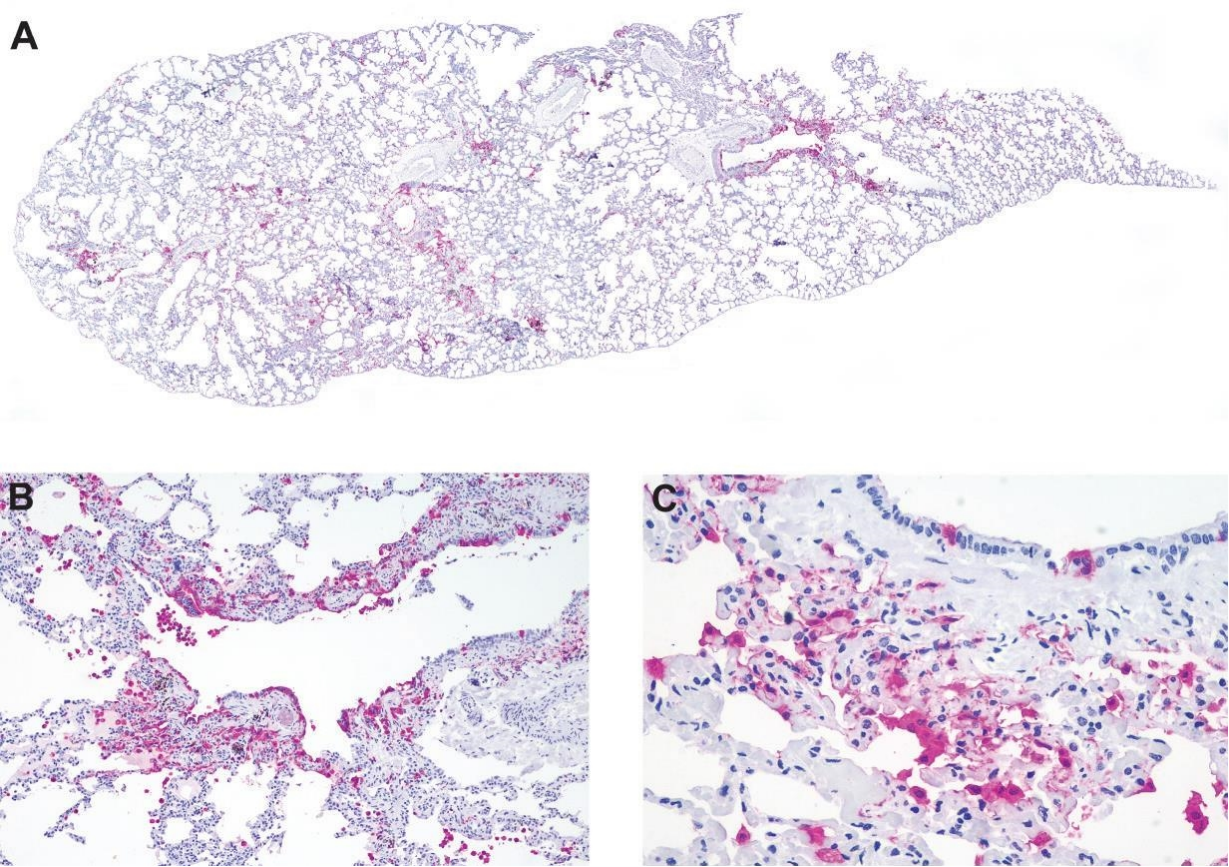
Anticanine distemper virus immunohistochemistry of lungs from free-ranging black-tufted marmosets (*Callithrix penicillata*) naturally infected with the canine distemper virus. (A) Lung, multifocal areas of immunostained cells. Magenta chromogen, subgross view. (B) Lung, bronchi mucosa with multifocal to coalescent immunostained epithelial cells and multifocal immunostained alveolar macrophages. Magenta chromogen, 100x. (C) Lung, alveoli with multifocal to coalescent immunostained type I pneumocytes and alveolar macrophages. Magenta chromogen, 400x.

Lungs from all marmosets were positive for CDV antigens (Table 6). Bronchial and bronchiolar epithelium (Figure 6A,B), type I pneumocytes, and alveolar macrophages (Figure 6C) were positive in all marmosets (7/7) ranging from a few to moderate numbers of positive cells. Trachea was evaluated in four marmosets and in all of them the epithelial cells and the connective tissue of the lamina propria were positive (4/4).

**Figure 7:**
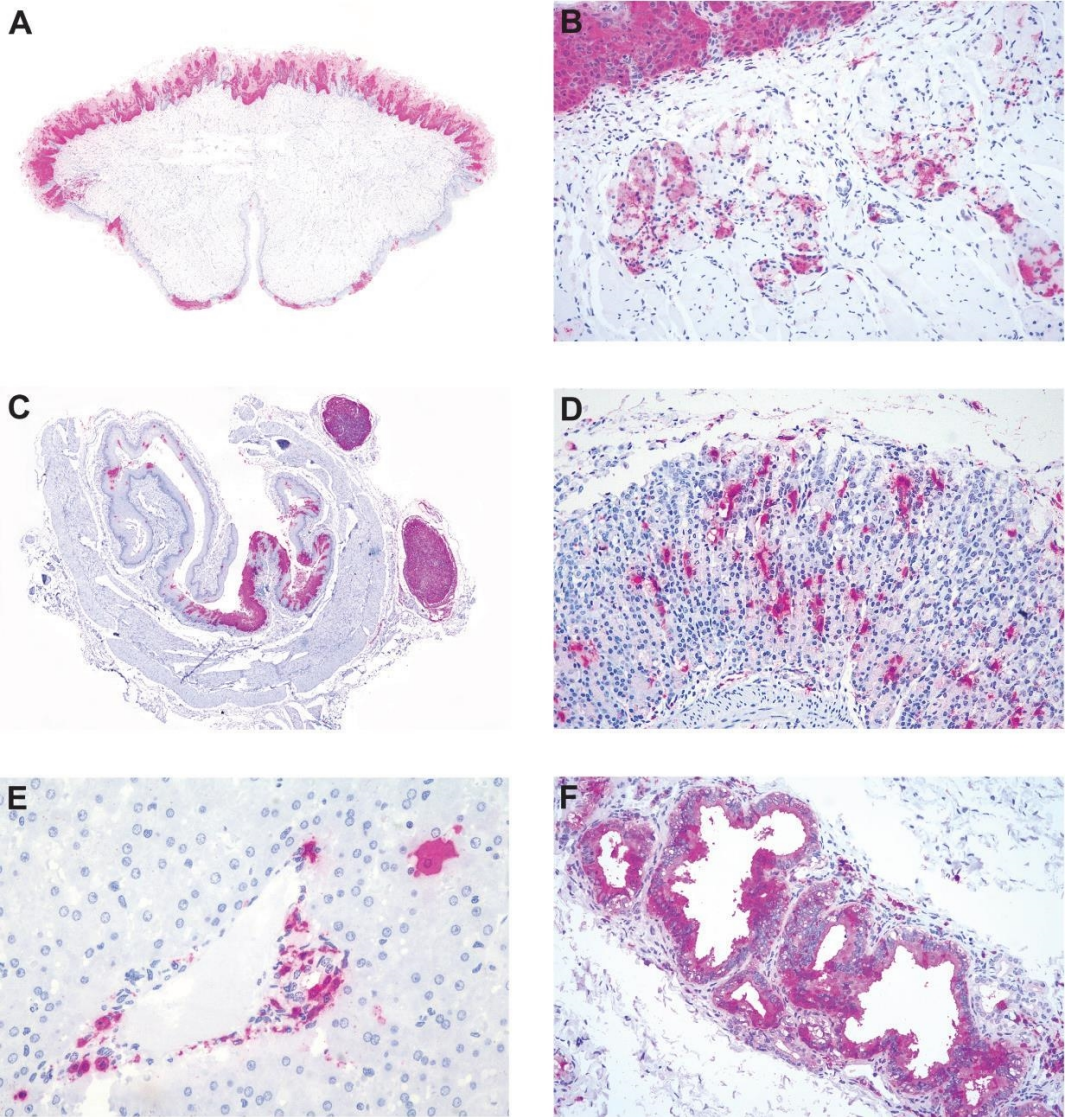
Anti-canine distemper virus immunohistochemistry of digestive tract organs from free-ranging black-tufted marmosets (*Callithrix penicillata*) naturally infected with the canine distemper virus. (A) Tongue, mucosa with multifocal to coalescent immunostained epithelial cells. Magenta chromogen, subgross view. (B) Tongue, lingual salivary glands with multifocal to coalescent immunostained epithelial cells. Magenta chromogen, 200x. (C) Esophagus, mucosa with multifocal to coalescent immunostained epithelial cells and lymph nodes with diffuse immunostained cells. Magenta chromogen, suggross view. (D) Stomach, mucosa with multifocal immunostained epithelial cells. Magenta chromogen, 200x. (E) Liver, multifocal immunostained hepatocytes, bile duct epithelial cells, and Kupffer cells. Magenta chromogen, 400x. (F) Gallbladder, mucosa with diffuse immunostained epithelial cells and lamina propria with multifocal immunostained cells. Magenta chromogen, 200x.

Samples of the digestive system were also evaluated (Table 7). Tongue, liver, and gallbladder from all marmosets were evaluated, however other organs, particularly the stomach and intestines, were not evaluated in all marmosets. Tongue epithelium was positive for CDV in all marmosets (7/7) (Figure 7A). Immunostaining was also positive in the subepithelial connective tissue (7/7) and in the lingual salivary glands (5/5) (Figure 7B). Epithelial cells were also immunopositive in the mucosa of the esophagus (4/4) (Figure 7C), stomach (4/4) (Figure 7D), small intestine (1/3) and large intestine (3/4). Rectum was evaluated only in the case of marmoset 2. CDV antigens were detected multifocally in superficial mucosal epithelial cells and mucosal glands.

Liver and gall bladder were immunopositive in all marmosets (7/7). Mild to moderate numbers of hepatocytes were positive in four of the seven marmosets, and biliary ducts (5/7) and Kupffer cells (6/7) were also positive (Figure 7E). Gall bladder epithelium was positive in all seven marmosets (7/7) (Figure 7F), even in individuals with more advanced autolysis.

**Figure 8:**
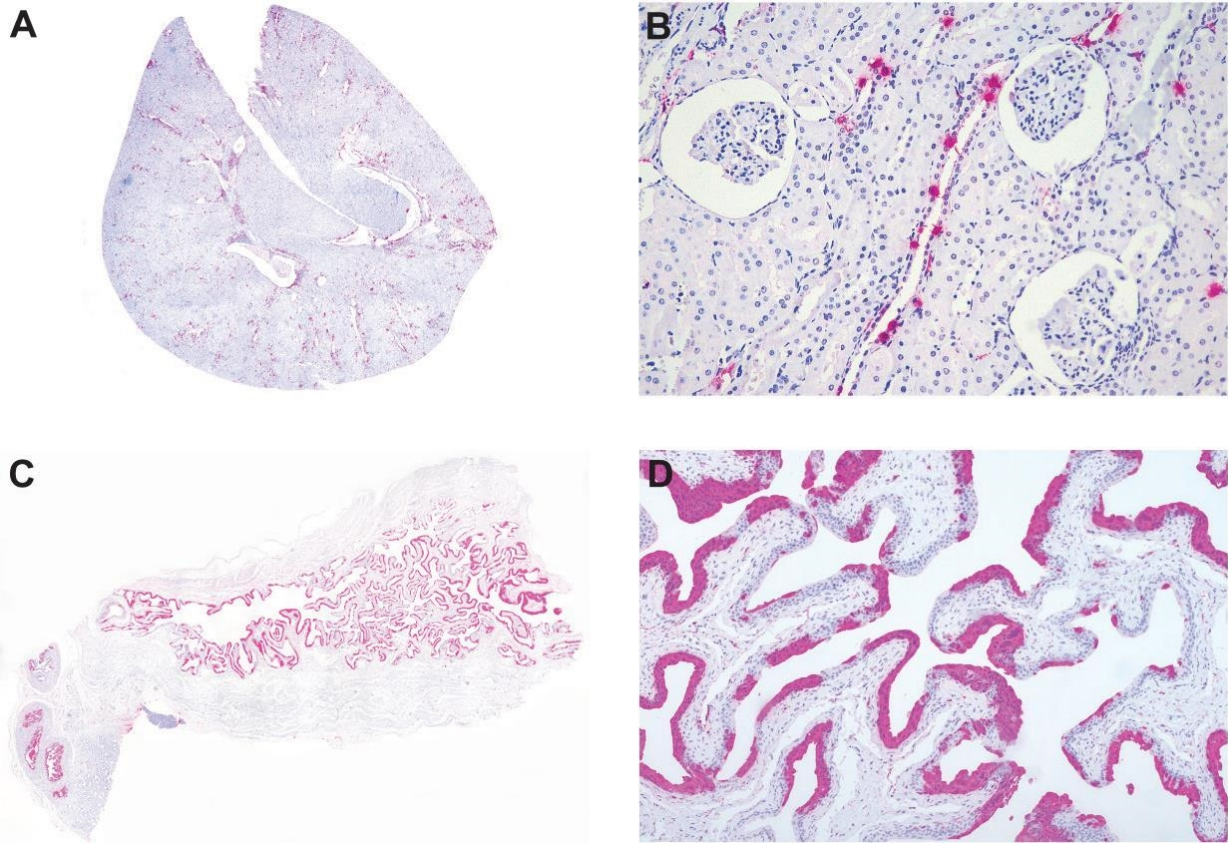
Anticanine distemper virus immunohistochemistry of urinary tract organs from free-ranging black-tufted marmosets (*Callithrix penicillata*) naturally infected with the canine distemper virus. (A) Kidney, multifocal areas of immunostained cells. Magenta chromogen, subgross view. (B) Kidney, tubules with multifocal immunostained epithelial cells. Magenta chromogen, 200x. (C) Urinary bladder, mucosa with diffuse immunostained epithelial cells and prostate with multifocal to coalescent immunostained epithelial cells. Magenta chromogen, subgross view. (D) Urinary bladder, mucosa with multifocal to coalescent immunostained epithelial cells. Magenta chromogen, 100x.

Kidneys were positive in all tested marmosets (6/6) (Table 8 and Figure 8). Antigens were detected in epithelial cells in both cortical and medullary tubules (6/6 and 4/5, respectively) (Figure 8B), glomeruli (4/6), interstitium (5/6), and the urothelial cells of the pelvis (4/4). Additionally, urothelial cells of the urinary bladder tested positive in all marmosets from which urinary bladder was sampled (5/5) (Figure 8C,D).

**Figure 9:**
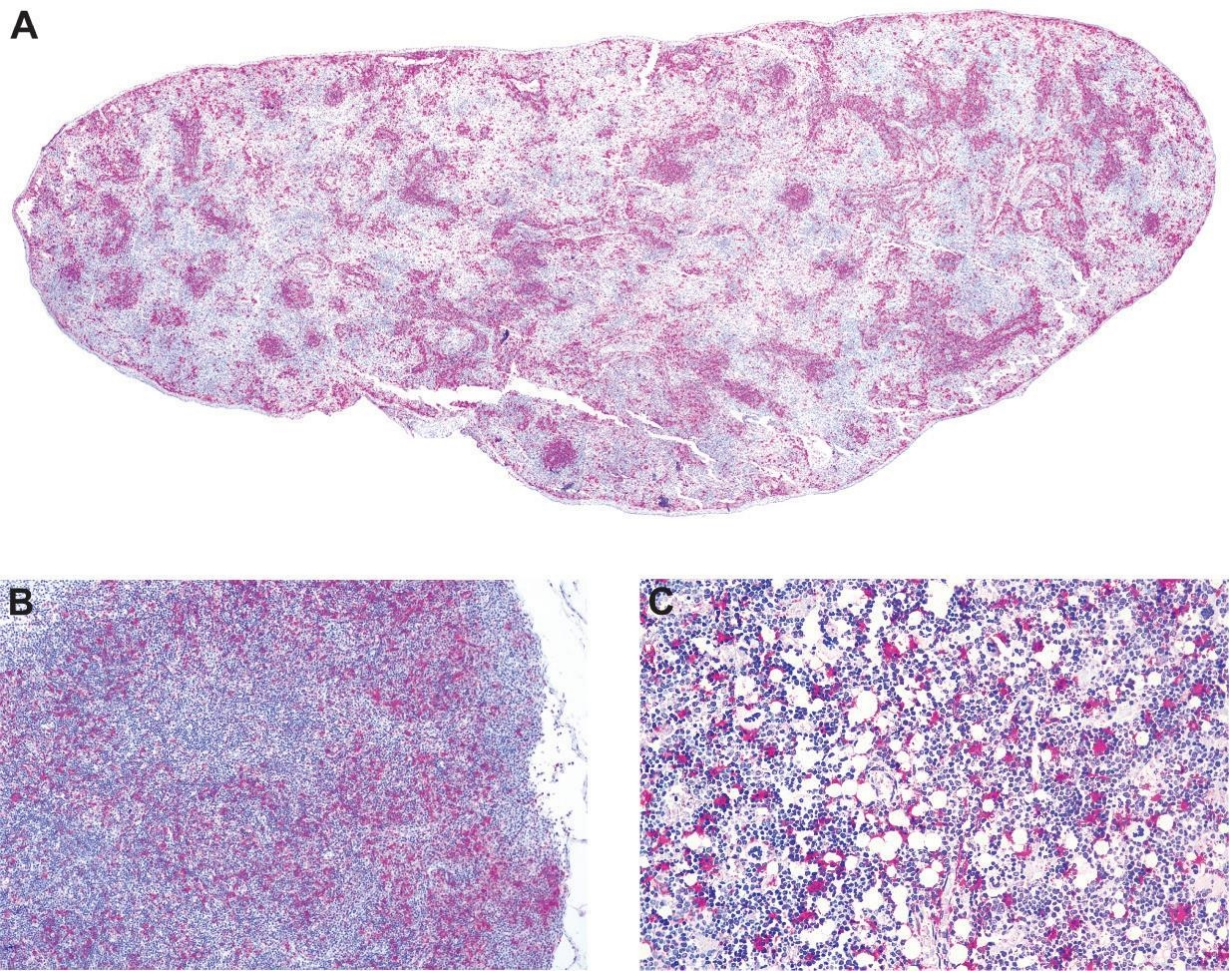
Anticanine distemper virus immunohistochemistry of lymphoid organs from free-ranging black-tufted marmosets (*Callithrix penicillata*) naturally infected with the canine distemper virus. (A) Spleen, white pulp with multifocal to coalescent immunostained cells. Magenta chromogen, subgross view. (B) Lymph node, lymphoid tissue with multifocal to coalescent immunostained cells. Magenta chromogen, 100x. (C) Bone marrow, multifocal to coalescent immunostained cells. Magenta chromogen, 200x.

Lymphoid organs (spleen, lymph nodes, and bone marrow) were consistently positive for CDV antigens, with all marmosets positive in all three organs (Table 9). Antigens were found in both the white and red pulp of the spleen (Figure 9A). In lymph nodes, antigens were mainly associated with lymphoid follicles (Figure 9B). CDV antigens were found in both erythroid and myeloid cells of the bone marrow in all marmosets (7/7) (Figure 9C); however, only a few megakaryocytes were positive in three marmosets.

**Figure 10:**
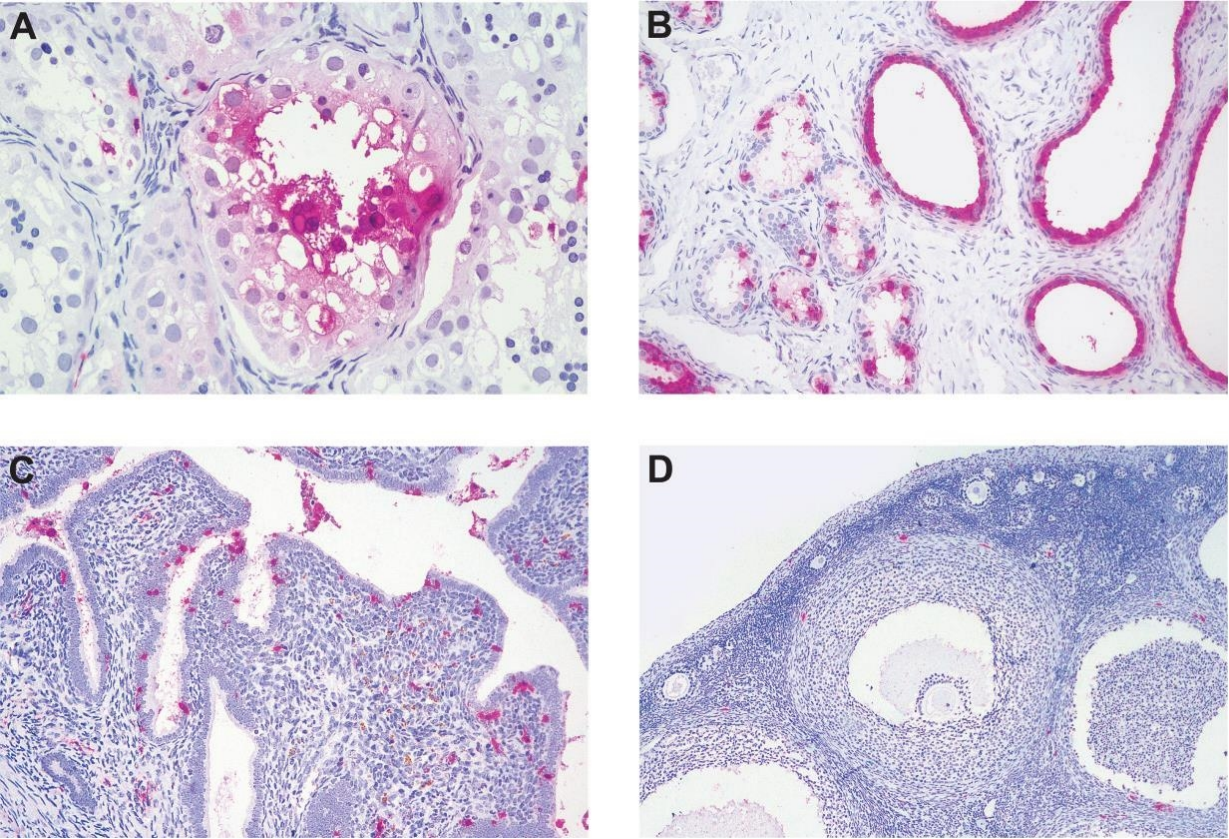
Anticanine distemper virus immunohistochemistry of male (A and B) and female (C and D) reproductive organs from free-ranging black-tufted marmosets (*Callithrix penicillata*) naturally infected with the canine distemper virus. (A) Testicle, seminiferous tubules with multifocal to coalescent immunostained spermatogenic and Sertoli cells. Magenta chromogen, 400x. (B) Epididymis, tubules with multifocal to diffuse immunostained epithelial cells. Magenta chromogen, 200x. (C) Uterus, endometrium with multifocal immunostained superficial epithelial and stromal cells. Magenta chromogen, 200x. (D) Ovary, multifocal immunostained stromal cells. Magenta chromogen, 200x.

Both male (2/2) and female (3/3) reproductive organs were positive for CDV antigens (Table 10). Testes, epididymides, and prostate glands of two tested marmosets were positive (2/2). Spermatogenic cells (spermatogonia, spermatocytes, and spermatids), Sertoli cells, and Leydig cells were positive (2/2) (Figure 10A). Epithelial cells of the epididymis (Figure 10B), prostate and prostatic urethra were positive for CDV antigens. In females, epithelial cells of the endometrium (Figure 10C) and vagina, as well as the endometrial glands were positive in all of them (3/3). In the ovaries, CDV antigens were more consistently detected in the stroma (3/3) (Figure 10D); however, antigens were also detected on the mesothelial surface of the ovary (germinal epithelium) (1/3) and luteal cells (2/2).

**Figure 11:**
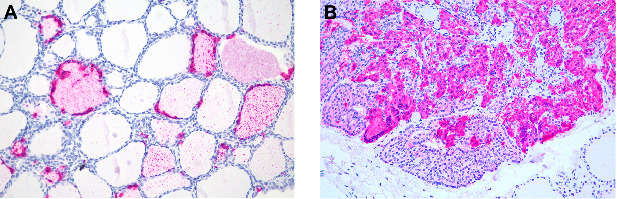
Anticanine distemper virus immunohistochemistry of thyroid glands (A) and parathyroid glands (B) from free-ranging black-tufted marmosets (*Callithrix penicillata*) naturally infected with the canine distemper virus. (A) Thyroid gland, follicles with multifocal to coalescent immunostained cells and multifocal pinpoint immunostaining of the colloid. Magenta chromogen, 200x. (B) Parathyroid gland, multifocal to coalescent immunostained chief cells, including multinucleated syncytial cells. Magenta chromogen, 200x.

Other organs were also evaluated (Table 11). All evaluated thyroid glands were positive in both follicular cells and colloid (6/6 for both) (Figure 11A). Parathyroid glands were present in only three of the six evaluated thyroid glands, and all of them were positive for CDV antigens (3/3) (Figure 11B). Pancreas from two marmosets tested positive with antigens mostly detected in the exocrine cells (2/2). CDV antigens were found in both cortical (6/7) and medullary cells (4/7) of the adrenal glands. In the heart, only a few cells, mainly endothelial cells or fibroblasts in the connective tissue of the endocardium, myocardium, and epicardium, were antigen-positive in all marmosets (6/6).

## 4. Discussion

CDV, like other morbilliviruses, is a lymphotropic, epitheliotropic, and neurotropic and is a highly immunosuppressive pathogen [2]. For morbilliviruses to establish in a population, the host species must not only be susceptible, but effective direct contacts must also occur [26, 27]. We described a lethal outbreak of CDV in naturally infected black tufted marmosets.

The primary source of infection for the primates in the outbreak remains unknown. This was considered an outbreak because of the abnormal and exceedingly high number of sick and dead marmosets in a forested area in a local zoo over the period of 1 month. Due to the frequent movement of these animals to areas outside the zoo, it is possible that they came into contact with stray dogs during their viral shedding period or through interaction with other infected wild animals. The outbreak could also have originated from contact with contaminated secondary sources such as water or food. During this outbreak in free-ranging marmosets, no animal kept at the zoo and no other species of local wildlife was diagnosed with CDV, favoring the hypothesis that the outbreak was limited to the free-ranging population of marmosets. However, the possibility of asymptomatic viral shedding by another animal cannot be ruled out.

The seven marmoset outbreak sequences of CDV displayed high pairwise identity (99.1%), indicating close genetic relatedness among the samples. Phylogenetic analysis placed them as a sister group to the Europe/South American 1 genotype, which includes sequences from domestic animals, wild mammals, and the 5804-vaccine strain. Based on phylogenetic analysis, all seven CDV-positive marmosets reported here shared a closely related CDV strain, supporting the theory that the animals had contact with the same source of infection or the virus spread among marmosets from the same group after an introduction. Although the sequenced fragment is relatively short to allow definitive conclusions, the marmoset sequences showed higher identity to lab-cultured 5804P and 5804 lab-cultured vaccine strains than to sequences from domestic dogs or wild mammals within the Europe/South American 1 genotype. The two closest sequences to those identified in this study correspond to the 5804 vaccine strain, a wild type CDV adapted to grow in Vero cells for vaccine production purposes [28].

Adaptation to Vero cells, a cell line derived from African green monkeys (*Cercopithecus aethiops*) kidney cells [29] may support the hypothesis that passages in cultured primate cells may favor host adaptation to other primate species. A recent study demonstrated that successive passages of CDV in Vero cells expressing the human SLAM (CD150)—a receptor for morbilliviruses in various host species—resulted in a CDV strain carrying a point mutation in the H gene. Importantly, this spontaneously mutated strain was capable of infecting leukocytes [30]. These findings support the hypothesis of a potential risk for human adaptation of CDV. However, further genomic studies are needed to identify or exclude mutations that may have resulted in primate adaptation of the CDV strain identified in this report. Importantly, in contrast to other morbilliviruses, which are quite host restricted, the ability of CDV to jump from one host species to another is well known [27]. Therefore, CDV must also be recognized as a threat to endangered wildlife, with the potential to drive population declines or even extinction of novel susceptible wild host species.

Populations of black-tufted marmosets (*C. penicillata*) are frequently found in urban areas in Brazil and have expanded beyond their natural distribution [16, 31]. The detection of CDV in this species, combined with their broad territorial range and interspecific interactions, creates conditions that may facilitate spillovers to other mammal species, including humans. This raises concerns about the impact on biodiversity conservation and public health [2, 4–6].

Clinical signs and gross lesions observed in this outbreak are similar to those caused by other viral diseases, underscoring the need to differentiate *Morbillivirus* infections from those of other viruses in primates. CDV in domestic dogs can induce neurological signs, including seizures, ataxia, paraparesis, or tetraparesis, and mainly myoclonus [32]. Interestingly, generalized myoclonus was observed in one of the CDV-infected marmosets (Supporting Information 1: Video S1). In dogs, CDV-elicited myoclonus is associated with demyelination, which is one of the hallmarks of CDV infection in this species [33]. However, demyelination was not observed in any of the CDV-infected marmosets in this study.

Herpesvirus is an important differential diagnosis in neurological disorders of neotropical primates. This is of particular concern in urban environments where exposure to human waste that may be infected with human herpesviruses is frequent [34]. Due to the neurological manifestations associated with HSV-1 infection, it is important to consider HSV-1 testing in neotropical primates that present with neurological symptoms [35]. In the same context, rabies should also be included as a crucial differential diagnosis, given its zoonotic potential and the overlap of neurological signs with other viral infections [36].

The ability of CDV to infect species other than its preferred canid hosts demonstrates its capacity to infect a wide range of species, highlighting the evolutionary potential of this highly infectious virus and making it one of the most dangerous members of the *Morbillivirus* genus [26].

HSV-1 infection, which is characterized by vesicular and ulcerative oral, periocular, and nasal lesions, along with conjunctivitis, lethargy, anorexia, and ataxia in neotropical primates [37]. Based on the current study, CDV infection can induce neurological and cutaneous signs similar to those observed in HSV-1-infected marmosets, suggesting that CDV infection may have been misdiagnosed and underreported in other instances. However, cutaneous lesions in the CDV-positive marmosets reported here are proliferative, which differs from HSV-1 cutaneous lesions, which are mainly ulcerative [38].

In dogs and susceptible wild mammals, CDV causes long-lasting and profound inhibition of both cellular and humoral immune functions, leading to immunosuppression, lymphocyte loss, and leukopenia, which increases susceptibility to opportunistic infections [2, 3]. Due to immunosuppression, CDV infection is frequently associated with other infectious diseases, such as *Sarcocystis* sp., *Toxoplasma gondii*, and *Salmonella* sp. [39, 40]. CalHV-3 coinfection was also diagnosed in three marmosets in this study. CalHV-3, homologous to the Epstein-Barr Virus, typically does not cause severe disease during primary infection in NWP [41]. However, it may remain latent and immunosuppression or transmission to divergent species can lead to viral-induced lymphoproliferation, potentially progressing to lethal lymphoma [42]. The absence of lesions compatible with CalHV-3 and the detection of CDV through IHC and molecular methods support the hypothesis that CDV-induced immunosuppression may have reactivated latent CalHV-3 infections in affected marmosets.

Lesions associated with CDV infection in this outbreak mainly affected skin and brain. Skin lesions were proliferative with acanthosis and hyperkeratosis, which is also described mainly in footpads and nasal planum (so-called “hard pad disease”) of CDV-infected domestic dogs [43, 44]; however, it has also been described in other locations such as periocular skin, scrotum, and vulva [43]. In naturally infected raccoons (*Procyon lotor*) in one study, cutaneous lesions included dermatitis, folliculitis, and ulceration [45]. Additionally, in the same study epithelial syncytial cells in raccoons were considered rare, which differed from the marmosets in this report, in which syncytial cells were frequently found not only in the skin but also in the epithelial cells of other organs. Hyperkeratosis has also been described in a CDV-positive giant anteater (*Myrmecophaga tridactyla*) [40], in a captive CDV-positive giant panda (*Ailuropoda melanoleuca*) with lesions affecting the nose and footpads [46] and in captive civets (Asian palm civets [*Paradoxurus hermaphrodites*, *n* = 18]; a masked palm civet [*Paguma larvata*, *n* = 1], and a small Indian civet [*Viverricula indica*, *n* = 1]) with lesion in the nose, muzzle, and footpads [47].

In the cases reported here, brain lesions were mild and characterized mainly by gliosis with gemistocytes and Alzheimer type II cells, neuronal necrosis, meningitis, choroiditis. and intranuclear eosinophilic inclusion bodies in neurons and astrocytes, which differ from the typical lesions observed in domestic dogs [33]. In domestic dogs, neurologic lesions are often characterized by demyelination, which was not observed in marmosets in this study. For other nondomestic species histological lesions are variable. In one naturally infected Japanese monkey (*Macaca fuscata*) the main neurologic lesions were nonsuppurative encephalitis, malacia, and intranuclear inclusion bodies mainly in glial cells [6]. Gliosis with gemistocytes was associated with CDV infection in sea otters [39]. Neuronal necrosis has also been described in naturally infected Southern tamandua (*Tamandua tetradactyla*) [48], black bears (*Ursus americana*) [49] and sea otters (*Enhydra lutris*) [39]. Inclusion bodies were reported as intracytoplasmatic and intranuclear in sea otters (*Enhydra lutris*) [39]. Neurologic lesions observed in free-ranging naturally infected red foxes (*Vulpes vulpes*), stone martens (*Martes foina*), and badgers were mainly gliosis; however, nonsuppurative meningoencephalitis and neuronal necrosis were also observed, and inclusion bodies were mainly intranuclear in neurons [11]. No specific neurologic histological lesions were reported in maned wolves (*Chrysocyon brachyurus*) and giant anteaters (*Myrmecophaga tridactyla*) [40, 50].

Here we also investigated antigen tissue distribution for several organs. All organs tested were positive for CDV antigens, which highlights the pantropic feature of CDV. Skin distribution of CDV antigens were previously characterized in domestic dogs and showed a similar profile to the observed in marmosets with CDV antigens found in the epidermis, hair follicles, apocrine, and sebaceous glands, blood vessels and dermal connective tissue [43, 44]. CDV antigens were also identified in raccoons with a similar distribution [45]. Characterization of CDV distribution can be useful for developing noninvasive screening methods.

For other organs, CDV antigen tissues distribution is reported in smaller studies and case reports; however, published information corroborates with our findings in marmostes. CDV antigens wasdetected in the cytoplasm and nuclei of neurons and glial cells of one naturally infected Japanese monkey (*Macaca fuscata*) [6] and from free-ranging red foxes, stone martens, and badgers (*Meles meles*) [11]. In captive civets, CDV antigens were described only in glial cells [47]. Antigens were described in the epithelial cells of the tongue of Linnaeus's 2-toed sloths (*Choloepus didactylus*) [51] and captive civets [47]. In the liver, CDV antigens were described in the hepatocytes of Linnaeus's 2-toed sloths (*Choloepus didactylus*) [51] and captive civets, in which bile ducts epithelial cells were also positive for CDV antigens [47].

The SLAM is the primary receptor for morbilliviruses, and host specificity and transmission are related to significant differences between SLAM receptors in different species [1, 52]. In contrast, nectin-4, another receptor with a highly conserved amino acid sequence across different mammals, reduces host specificity [3, 52]. Although our comprehension of the basis for adaptation of CDV to novel host species, the following aspects should be considered: (i) There is a high level of similarity in SLAM and nectin-4 sequences between monkeys and humans [1, 3, 52]; (ii) isolates from recent primate outbreaks suggest that mutations in the viral H protein, favored by frequent viral passages, may contribute to the adaptation of CDV strains from monkeys to humans [1, 3, 27]; (iii) reduction in measles vaccine adherence may facilitate CDV adaptation to humans [53, 54]; and (iv) infections by different morbilliviruses may induce partial cross-protection, and large outbreaks of CDV pose a risk to animals without immunity to morbilliviruses [15]. Each of these factors, along with the detection of CDV-associated lethal disease in neotropical primates, raises significant concerns about possible spillover of this virus from marmosets to humans, and underscores the need for a One Health approach that includes CDV testing in primate surveillance programs.

The detection of CDV-associated lethal disease in neotropical primates raises significant concerns about the potential for future spillovers of this virus and underscores the need for a One Health approach, as well as the inclusion of CDV in primate surveillance programs.

## 5. Conclusion

This study demonstrated that CDV is capable of infecting free-ranging black-tufted marmosets and that infection can lead to neurological signs, cutaneous lesions, and lethal outcomes. Viral genetic material and antigens were detected in several organs, indicating a pantropic distribution of CDV in this neotropical primate species. CDV sequences identified here were closely related to Vero cell-adapted CDV strains. The detection of CDV-associated lethal disease in neotropical primates raises concerns about potential spillover events and highlights the importance of a One Health approach and the inclusion of CDV in primate surveillance programs.

## Figures and Tables

**Table 1:** Histological lesion in black-tufted marmosets (*Callithrix penicillata*) naturally infected with the canine distemper virus.

ID	Date	Sex/age	Histopathology
01	June 2, 2022	Male/adult	Skin: severe diffuse acanthosis with mild multifocal syncytial cells, moderate multifocal epithelial degeneration, multifocal severe orthokeratotic hyperkeratosis, multifocal moderate follicular keratosis, and moderate multifocal neutrophilic dermatitis Tongue: mild multifocal epithelial syncytial cells Brain: severe multifocal gliosis with gemistocytes, syncytial cells, and Alzheimer type 2 astrocytes, rare intranuclear eosinophilic inclusion bodies Lungs: moderate multifocal lymphocytic and neutrophilic interstitial pneumonia, severe multifocal atelectasis, and moderate locally extensive intralveolar hemorrhage Liver: mild multifocal lipidosis and mild multifocal glycogenosis Gallbladder: mild multifocal epithelial syncytial cells Spleen: severe diffuse lymphoid depletion, mild diffuse congestion, moderate multifocal extramedullary hematopoiesis Lymph nodes: severe diffuse lymphoid depletion, mild multifocal hemosiderosis, and mild multifocal extramedullary hematopoiesis Testes: severe diffuse degeneration Urinary bladder: rare multifocal epithelial syncytial cells Prostate: moderate locally extensive neutrophilic interstitial prostatitis Parathyroid gland: mild multifocal syncytial cells

02	June 10, 2022	Female/adult	Skin: severe diffuse acanthosis with moderate multifocal syncytial cells, mild multifocal epithelial degeneration, severe multifocal orthokeratotic and parakeratotic hyperkeratosis, multifocal moderate follicular keratosis, and multifocal moderate neutrophilic dermatitis Tongue: mild multifocal epithelial syncytial cells Brain: mild multifocal lymphocytic and neutrophilic meningitis, moderate diffuse lymphocytic and neutrophilic coroiditis, mild focal gliosis with neuronal necrosis Lungs: mild multifocal neutrophilic interstitial pneumonia Liver: mild multifocal lipidosis Spleen: moderate diffuse lymphoid depletion, moderate diffuse congestion, mild multifocal hemosiderosis Lymph nodes: severe diffuse lymphoid depletion and mild multifocal hemosiderosis Parathyroid gland: moderate multifocal syncytial cells Esophagus: mild multifocal epithelial syncytial cells

03	June 17, 2022	Female/adult	Skin: mild focal neutrophilic ulcerative dermatitis with rare multifocal epithelial syncytial cells, mild multifocal orthokeratotic hyperkeratosis, and mild multifocal crusts Tongue: rare multifocal epithelial syncytial cells Brain: moderate multifocal gliosis with Alzheimar type 2 astrocytes Lungs: locally extensive atelectasia and mild multifocal alveolar edema Liver: mild multifocal neutrophilic and necrotic hepatitis, mild diffuse lipidosis, moderate multifocal hemorrhage Gallbladder: intraluminal severe hemorrhage with mild focal cholelith and mild trematode eggs Spleen: moderate diffuse lymphoid depletion, moderate diffuse congestion, moderate multifocal extramedullary hematopoiesis, mild multifocal hemosiderosis Lymph nodes: moderate diffuse lymphoid depletion, mild extramedullary hematopoiesis, and mild multifocal hemosiderosis Parathyroid gland: mild multifocal syncytial cells

04	June 26, 2022	Male/adult	Skin: moderate multifocal acanthosis with rare multifocal syncytial cells, moderate multifocal epithelial degeneration, moderate multifocal orthokeratotic hyperkeratosis, multifocal cellular crusts and moderate multifocal neutrophilic dermatitis Tongue: mild multifocal syncytial cells Brain: mild focal lymphocytic meningitis, moderate multifocal gliosis with Alzheimer type 2 astrocytes, gemistocytes, focal neuronal necrosis, mild multifocal intranuclear eosinophilic inclusion bodies Lungs: moderate multifocal atelectasia, mild multifocal alveolar hemorrhage Liver: mild multifocal lipidosis Spleen: mild lymphoid depletion, mild multifocal hemosiderosis, mild multifocal extramedullary hematopoiesis Lymph nodes: mild diffuse lymphoid depletion, mild multifocal hemosiderosis Parathyroid gland: moderate multifocal syncytial cells Testes: moderate diffuse degeneration

05	July 6, 2022	Male/adult	Skin: moderate diffuse acanthosis with moderate multifocal epithelial syncytial cells, moderate multifocal epithelial degeneration, moderate multifocal parakeratotic hyperkeratosis, multifocal cellular crusts, multifocal moderate follicular keratosis. Mild multifocal neutrophilic and histiocytic dermatitis Tongue: moderate multifocal epithelial syncytial cells Brain: mild focal lymphoplasmacytic meningitis, mild multifocal gliosis with Alzheimer type 2 astrocytes and neuronal necrosis Lungs: moderate multifocal neutrophilic interstitial pneumonia Liver: moderate multifocal necrosis with fibrin and hemorrhage, mild portal fibrosis with bile duct proliferation Spleen: moderate diffuse lymphoid depletion, moderate diffuse congestion, moderate multifocal extramedullary hematopoiesis Lymph nodes: moderate diffuse lymphoid depletion, mild multifocal extramedullary hematopoiesis, mild multifocal hemosiderosis Parathyroid gland: moderate multifocal syncytial cells Testes: moderate diffuse degeneration and mild multifocal to coalescent histiocytic and neutrophilic orchitis

06	July 6, 2022	Female/adult	Skin: mild diffuse orthokeratotic hyperkeratosis Brain: mild diffuse congestion Lungs: moderate multifocal lymphohistiocytic, plasmacytic and neutrophilic interstitial pneumonia, moderate multifocal edema Liver: mild diffuse lipidosis Spleen: mild diffuse lymphoid depletion, moderate diffuse congestion, moderate multifocal extramedullary hematopoiesis Lymph nodes: moderate lymphoid depletion, moderate multifocal hemorrhage, mild multifocal hemosiderosis Parathyroid gland: mild multifocal syncytial cells

07	July 11, 2022	Male/adult	Skin: mild diffuse orthokeratotic hyperkeratosis with mild multifocal cellular crusts Tongue: rare multifocal syncytial cells Lungs: moderate multifocal alveolar edema and hemorrhage Liver: mild diffuse lipidosis Lymph nodes: mild multifocal hemosiderosis

**Table 2:** Molecular investigation (canine distemper virus, measles, *Lyssavirus*, and pan-herpesvirus) in black-tufted marmosets (*Callithrix penicillata*) naturally infected with the canine distemper virus.

ID	Sample	RT-PCR CDV	RT-PCR measles	RT-PCR *Lyssavirus*	NESTED-PCR PAN-HERPES	GenBank accession number
01	Oral swab	Pos	Neg	NP	Neg	CDV_BR_01 PQ356990
	Rectal swab	Neg	Neg	NP	NP	NA
	Brain	Pos	Neg	Neg	Pos	CalHV-3_01_BR PQ362650
	Skin	Pos	Neg	NP	Neg	NA
	Lungs	Pos	Neg	NP	NP	NA
	Spleen	Pos	Neg	NP	NP	NA
	Liver	Pos	Neg	NP	NP	NA
	Kidneys	Pos	Neg	NP	NP	NA

02	Oral swab	NS	NS	NP	NS	NA
	Rectal swab	NS	NS	NP	NS	NA
	Brain	Pos	Neg	Neg	Neg	NA
	Skin	Pos	Neg	NP	Neg	NA
	Lungs	Pos	Neg	NP	NP	CDV_BR_02 PQ356991
	Spleen	Pos	Neg	NP	NP	NA
	Liver	Pos	Neg	NP	NP	NA
	Kidneys	Pos	Neg	NP	NP	NA

03	Oral swab	NS	NS	NP	NS	NA
	Rectal swab	NS	NS	NP	NS	NA
	Brain	Pos	Neg	Neg	Neg	NA
	Skin	Pos	Neg	NP	Neg	NA
	Lungs	Pos	Neg	NP	NP	CDV_BR_03 PQ356992
	Spleen	Pos	Neg	NP	NP	NA
	Liver	Pos	Neg	NP	NP	NA
	Kidneys	Pos	Neg	NP	NP	NA

04	Oral swab	Pos	Neg	NP	Neg	CDV_BR_04 PQ356993
	Rectal swab	Pos	Neg	NP	NP	NA
	Brain	Pos	Neg	Neg	Pos	NA
	Skin	Pos	Neg	NP	Pos	NA
	Lungs	Pos	Neg	NP	NP	NA
	Spleen	Pos	Neg	NP	NP	NA
	Liver	Pos	Neg	NP	NP	NA
	Kidneys	Pos	Neg	NP	NP	NA

05	Oral swab	NS	NS	NP	NS	NA
	Rectal swab	NS	NS	NP	NS	NA
	Brain	Pos	Neg	Neg	Neg	NA
	Skin	Pos	Neg	NP	Neg	CDV_BR_05 PQ356994
	Lungs	Pos	Neg	NP	NP	NA
	Spleen	Pos	Neg	NP	NP	NA
	Liver	Pos	Neg	NP	NP	NA
	Kidneys	Pos	Neg	NP	NP	NA

06	Oral swab	NS	NS	NP	NS	NA
	Rectal swab	NS	NS	NP	NS	NA
	Brain	Pos	Neg	Neg	Neg	NA
	Skin	Pos	Neg	NP	Neg	NA
	Lungs	Pos	Neg	NP	NP	CDV_BR_06 PQ356995
	Spleen	Pos	Neg	NP	NP	NA
	Liver	Pos	Neg	NP	NP	NA
	Kidneys	Pos	Neg	NP	NP	NA

07	Oral swab	NS	NS	NP	NS	NA
	Rectal swab	NS	NS	NP	NS	NA
	Brain	Pos	Neg	Neg	Pos	NA
	Skin	Pos	Neg	NP	Neg	CDV_BR_07 PQ356996
	Lungs	Pos	Neg	NP	NP	NA
	Spleen	Pos	Neg	NP	NP	NA
	Liver	Pos	Neg	NP	NP	NA
	Kidneys	Neg	Neg	NP	NP	NA

*Note:* All PCR reactions included negative controls lacking the template DNA, which did not yield any product.

Abbreviations: NA, not applicable; Neg, negative result; NP, test not performed; NS, no sample collected; Pos, positive result.

**Table 3:** Anticanine distemper virus immunohistochemistry results of free-ranging black-tufted marmosets (*Callithrix penicillata*) naturally infected with the canine distemper virus.

Organ	ID							Total
	1	2	3	4	5	6	7	
Brain	Pos	Pos	Pos	Pos	Pos	Pos	Pos	7/7
Skin	Pos	Pos	Pos	Pos	Pos	Pos	Pos	7/7
Tongue	Pos	Pos	Pos	Pos	Pos	Pos	Pos	7/7
Esophagus	Pos	Pos	Pos	Pos	NE	NE	NE	4/4
Stomach	Pos	Pos	Pos	Pos	NE	NE	NE	4/4
Small intestine	Pos	NE	NE	Pos	Pos	NE	NE	3/3
Large intestine	Pos	Pos	NE	Pos	Pos	NE	NE	4/4
Liver	Pos	Pos	Pos	Pos	Pos	Pos	Pos	7/7
Gallbladder	Pos	Pos	Pos	Pos	Pos	Pos	Pos	7/7
Trachea	NE	Pos	Pos	Pos	Pos	NE	NE	4/4
Lungs	Pos	Pos	Pos	Pos	Pos	Pos	Pos	7/7
Kidney	Pos	Pos	Pos	Pos	NE	Pos	Pos	6/6
Urinary bladder	Pos	Pos	Pos	Pos	NE	Pos	NE	5/5
Testes	Pos	Female	Female	Pos	NE	Female	NE	2/2
Prostate	Pos	Female	Female	Pos	NE	Female	NE	2/2
Ovaries	Male	Pos	Pos	Male	Male	Pos	Male	3/3
Uterus	Male	Pos	Pos	Male	Male	Pos	Male	3/3
Vagina	Male	Pos	Pos	Male	Male	Pos	Male	3/3
Lymph nodes	Pos	Pos	Pos	Pos	Pos	Pos	Pos	7/7
Spleen	Pos	Pos	Pos	Pos	Pos	Pos	Pos	7/7
Bone marrow	Pos	Pos	Pos	Pos	Pos	Pos	Pos	7/7
Thyroid	Pos	Pos	Pos	Pos	Pos	Pos	NE	6/6
Parathyroid	NP	NP	Pos	NP	Pos	Pos	NE	3/3
Adrenal	Pos	Pos	Pos	Pos	Pos	Neg	Pos	7/7

Abbreviations: NE, not evaluated; Neg, negative; NP, not present; Pos, positive.

**Table 4:** Tissues distribution of canine distemper virus antigens in brains of free-ranging black-tufted marmosets (*Callithrix penicillata*) naturally infected with the canine distemper virus.

Brain		ID							Total
		1	2	3	4	5	6	7^a^	
Telencephalic cortex	Neurons	0	0	++	+++	+++	+	NE	4/6
	Glial cells	+	0	++	++	++	++	NE	5/6
	Endothelial cells	+	+	+	++	+	0	NE	5/6
	Meninge	+	0	+	++	++	0	NE	4/6
	Choroid plexus	+	++	++	+	+	0^b^	NE	5/6
	Hippocampus	0	0	NE	NE	0	0	NE	0/4

Frontal lobe	Neurons	+++	++	0	++	++	++	NE	5/6
	Glial cells	+++	+	+	++	++	++	NE	6/6
	Endothelial cells	+	+	0	+	0	+	NE	4/6
	Meninge	+	0	+	+	+	+	NE	5/6

Occipital lobe	Neurons	0	0	0	+	0	0	NE	1/6
	Glial cells	+	0	+	+	++	+	NE	5/6
	Endothelial cells	+	+	+	+	+	0	NE	5/6
	Meninge	+	0	+	+	+	0	NE	4/6

Cerebelum	Molecular layer (neurons)	0	0	0	+	0	+	NE	2/6
	Purkinje cells (neurons)	0	0	0	0	0	0	NE	0/6
	Granular layer (neurons)	+	+	+	0	0	0	NE	3/6
	Glial cells	0	0	0	+	+	+	NE	3/6
	Endothelial cells	0	0	+	+	+	0	NE	3/6
	Meninge	+	0	0	+	+	0	NE	3/6
	Choroid plexus	++	+	0	0	++	0	NE	3/6

Brain stem	Neurons	0	0	0	+	+	0	NE	2/6
	Glial cells	+	+	+	++	++	+	NE	6/6
	Endothelial cells	+	+	+	+	+	0	NE	5/6
	Meninge	0	0	0	0	+	0	NE	1/6

*Note:* 0: negative; +: mild, focal or multifocal; ++: moderate, focal or multifocal; +++: intense multifocal to coalescent or diffuse.

Abbreviations: NE, not evaluated; NP, not present.

^a^Animal 7 had multiple brain lacerations due to trauma, making impossible to evaluate different segments of the brain; however, rare glial cells were positive.

^b^Evaluation harmed by autolysis.

**Table 5:** Tissues distribution of canine distemper virus antigens in the skin of free-ranging black-tufted marmosets (*Callithrix penicillata*) naturally infected with the canine distemper virus.

Skin		ID							Total
		1	2	3	4	5	6	7	
Face	Epidermis (epithelial cells)	+++	+++	+++	++	+++	+	NE^b^	6/6
	Sebaceous glands	++	++	++	+	++	++	NE^b^	6/6
	Apocrine glands	+	NP	+	NP	+	+	NE^b^	4/4
	Connective tissue (superficial dermis)	+++	++	++	++	++	+	NE^b^	6/6
	Endothelial cells	+	+	0	+	0	0	NE^b^	3/6

Scrotum	Epidermis (epithelial cells)	+++	NP^a^	NP^a^	+++	+++	NP^a^	NE^b^	3/3
	Sebaceous glands	++	NP^a^	NP^a^	+	++	NP^a^	NE^b^	3/3
	Apocrine glands	+	NP^a^	NP^a^	++	++	NP^a^	NE^b^	3/3
	Connective tissue	++	NP^a^	NP^a^	++	++	NP^a^	NE^b^	3/3
	Myocytes	+	NP^a^	NP^a^	0	0	NP^a^	NE^b^	1/3
	Endothelial cells	+	NP^a^	NP^a^	++	++	NP^a^	NE^b^	3/3

*Note:* 0: negative; +: mild, focal or multifocal; ++: moderate, focal or multifocal; +++: intense multifocal to coalescent or diffuse.

Abbreviations: NE, not evaluated; NP, not present.

^a^Female marmoset.

^b^Facial skin and scrotum not sampled, perianal and ear lob skin samples were both positive for CDV antigens on the epithelial cells of the epidermis and the connective tissue of the superficial dermis.

**Table 6:** Tissues distribution of canine distemper virus antigens in the respiratory system of free-ranging black-tufted marmosets (*Callithrix penicillata*) naturally infected with the canine distemper virus.

Respiratory system			ID							Total
			1	2	3	4	5	6	7	
Lungs	Bronchus/Bronchioli	Epithelial cells	+	+	++	++	++	+^a^	+	7/7
		Glands	0	0	0	0	0	0	0	0/7
		Lamina propria (connective tissue)	+	+	+	+	+	+	+	7/7
		Cartilage	0	0	0	0	0	0	0	0/0
	Alveoli	Pneumocytes (type I)	+	0	++	++	+	+	+	6/7
		Pneumocytes (type II)	NP	NP	NP	NP	NP	NP	NP	0/7
		Alveolar macrophages	+	+	++	++	++	+	+	7/7

Trachea		Epithelial cells	NE	+	+++	++	++	NE	NE	4/4
		Lamina propria (connective tissue)	NE	+	+	+	+	NE	NE	4/4
		Glands	NE	0	++	+	0	NE	NE	2/4
		Cartilage	NE	0	0	0	0	NE	NE	0/4
		Endothelial cells	NE	+	+	0	0	NE	NE	2/4

*Note:* 0: negative; +: mild, focal or multifocal; ++: moderate, focal or multifocal; +++: intense multifocal to coalescent or diffuse;.

Abbreviation: NE, not evaluated.

^a^Evaluation harmed by autolysis.

**Table 7:** Tissues distribution of canine distemper virus antigens in the digestive system of free-ranging black-tufted marmosets (*Callithrix penicillata*) naturally infected with the canine distemper virus.

Digestive system		ID							Total
		1	2	3	4	5	6	7	
Tongue	Epithelium	+++	+++	+++	++	+++	++	++	7/7
	Connective tissue	+	+	++	+	++	+	+	7/7
	Lingual salivary glands	++	++	NP	++	++	+	NP	5/5
	Myocytes	0	0	0	0	+	0	0	1/7
	Endothelial cells	0	0	0	0	0	0	0	0/7

Esophagus	Epithelium	++	++	++	++	NE	NE	NE	4/4
	Connective tissue	++	+	++	+	NE	NE	NE	4/4
	Myocytes	+	0	+	0	NE	NE	NE	2/4
	Endothelial cells	0	0	0	0	NE	NE	NE	0/4

Stomach	Mucosa (superficial epithelial cells)	+	0^a^	+	++	NE	NE	NE	3/4
	Chief and Parietal cells	+	++	+++	+++	NE	NE	NE	4/4
	Lamina propria	++	+	+	+	NE	NE	NE	4/4
	Submucosa (connective tissue)	+	+	+	0	NE	NE	NE	3/4
	Myocytes	0	+	+	0	NE	NE	NE	2/4
	Endothelial cells	0	0	+	0	NE	NE	NE	1/4

Small intestine	Mucosa (epithelial cells)	+	NE	NE	0^a^	0^a^	NE	NE	1/3
	Lamina propria	++	NE	NE	+	++	NE	NE	3/3
	Submucosa (connective tissue)	++	NE	NE	+	++	NE	NE	3/3
	Myocytes	+	NE	NE	+	+	NE	NE	3/3
	Endothelial cells	+	NE	NE	+	0	NE	NE	2/3

Large intestine	Mucosa (epithelial cells)	+	+^a^	NE	+	0	NE	NE	3/4
	Lamina propria	++	+	NE	+	+	NE	NE	4/4
	Submucosa (connective tissue)	+	0	NE	+	+	NE	NE	3/4
	Myocytes	0	0	NE	+	0	NE	NE	1/4
	Endothelial cells	0	0	NE	+	0	NE	NE	1/4

Liver	Hepatocytes	++	+	0	0	+	0	+	4/7
	Biliary ducts	+	+	+	0	+	+	0	5/7
	Kupfer cells	+	+	++	+	+	0	++	6/7
	Endothelial cells	0	+	+	0	+	0	0	3/7

Gallbladder	Epithelium	++	++^a^	++^a^	++^a^	++^a^	+^a^	++^a^	7/7
	Lamina propria	++	+^a^	+++	+	+	+	NP^a^	6/6

*Note:* 0: negative; +: mild, focal or multifocal; ++: moderate, focal or multifocal; +++: intense multifocal to coalescent or diffuse.

Abbreviations: NE, not evaluated; NP, not present.

^a^Evaluation harmed by autolysis.

**Table 8:** Tissues distribution of canine distemper virus antigens in the urinary system of free-ranging black-tufted marmosets (*Callithrix penicillata*) naturally infected with the canine distemper virus.

Urinary system		ID							Total
		1	2	3	4	5	6	7	
Kidney	Glomeruli	+	+	+	0	NE	0	+	4/6
	Tubules (cortex)	++	+	++	+	NE	+	+*⁣*^*∗*^	6/6
	Tubules (medulla)	+	+	++	+	NE	0	NP	4/5
	Connective tissue (interstitium)	++	+	+	+	NE	0	+	5/6
	Epithelial cells (pelvis)	+	+	+^a^	+++	NE	NP	NP	4/4
	Endothelial cells	+	+	+	0	NE	0	0	3/6

Urinary bladder	Epithelium	+++	+	++	+	NE	+^a^	NE	5/5
	Submucosa (connective tissue)	+	+	++	+	NE	+	NE	5/5
	Myocytes	0	+	0	0	NE	+	NE	2/5
	Endothelial cells	+	0	0	0	NE	+	NE	2/5

*Note:* 0: negative; +: mild, focal or multifocal; ++: moderate, focal or multifocal; +++: intense multifocal to coalescent or diffuse.

Abbreviations: NE, not evaluated; NP, not present.

^a^Evaluation harmed by autolysis.

**Table 9:** Tissues distribution of canine distemper virus antigens in lymphoid organs of free-ranging black-tufted marmosets (*Callithrix penicillata*) naturally infected with the canine distemper virus.

Lymphoid organs		ID							Total
		1	2	3	4	5	6	7	
Spleen	Capsule	+	+	+	+	+	+	0	6/7
	White pulp	+++	++	+++	+	+	+	+++	7/7
	Red pulp	++	+	+	+	+	+	++	7/7

Lymph node	Lymphoid follicles	+++	+++	+++	++	++	++	+++	7/7
	Subcapsular sinus	++	++	++	+	+	+	+	7/7
	Medullary sinus	++	++	++	+	+	+	+	7/7
	Capsule	+	+	+	+	+	+	+	7/7

Bone marrow	Erythroid and myeloid cells	+++	++	++	++	+	++	+^a^	7/7
	Megakaryocytes	+	0	+	+	0	0	0^a^	3/7

*Note:* 0: negative; +: mild, focal or multifocal; ++: moderate, focal or multifocal; +++: intense multifocal to coalescent or diffuse.

^a^Evaluation harmed by autolysis.

**Table 10:** Tissues distribution of canine distemper virus antigens in the reproductive system of free-ranging black-tufted marmosets (*Callithrix penicillata*) naturally infected with the canine distemper virus.

Reproductive system			ID							Total
			1	2	3	4	5	6	7	
Male	Testes	Spermatogenic and Sertoli cells	++^a^	NP^b^	NP^b^	++^a^	NE	NP^b^	NE	2/2
		Interstitium (Leydig cells and connective tissue)	+	NP^b^	NP^b^	+	NE	NP^b^	NE	2/2
	Epididymis	Epithelium	++	NP^b^	NP^b^	++	NE	NP^b^	NE	2/2
		Connective tissue	++	NP^b^	NP^b^	+	NE	NP^b^	NE	2/2
		Endothelial cells	+	NP^b^	NP^b^	+	NE	NP^b^	NE	2/2
	Prostate	Epithelium	+	NP^b^	NP^b^	++	NE	NP^b^	NE	2/2
		Connective tissue	+	NP^b^	NP^b^	+	NE	NP^b^	NE	2/2
		Prostatic urethra (epithelial cells)	+	NP^b^	NP^b^	+	NE	NP^b^	NE	2/2

Female	Ovary	Germinal epithelium	NP^c^	+	0	NP^c^	NP^c^	0	NP^c^	1/3
		Stroma	NP^c^	+	+	NP^c^	NP^c^	+	NP^c^	3/3
		Lutein cells	NP^c^	NP	+	NP^c^	NP^c^	+	NP^c^	2/2
		Follicles	NP^c^	0	0	NP^c^	NP^c^	0	NP^c^	0/3
	Uterus	Endometrium (superficial epithelium)	NP^c^	+	+++	NP^c^	NP^c^	NE^d^	NP^c^	2/2
		Endometrial glands	NP^c^	+	++	NP^c^	NP^c^	++	NP^c^	3/3
		Endometrial interstitium	NP^c^	+	++	NP^c^	NP^c^	+	NP^c^	3/3
		Myocytes (myometrium)	NP^c^	+	++	NP^c^	NP^c^	+	NP^c^	3/3
		Endothelial cells	NP^c^	0	0	NP^c^	NP^c^	0	NP^c^	0/3
	Vagina	Epithelium	NP^c^	++	+++	NP^c^	NP^c^	+	NP^c^	3/3
		Connective tissue	NP^c^	++	++	NP^c^	NP^c^	+	NP^c^	3/3
		Myocytes	NP^c^	0	+	NP^c^	NP^c^	0	NP^c^	1/3
		Endothelial cells	NP^c^	0	0	NP^c^	NP^c^	+	NP^c^	1/3

*Note:* 0: negative; +: mild, focal, or multifocal; ++: moderate, focal, or multifocal; +++: intense multifocal to coalescent or diffuse.

Abbreviations: NE, not evaluated; NP, not present.

^a^Including multinucleated cells.

^b^Female marmoset.

^c^Male marmoset.

^d^Evaluation harmed by autolysis.

**Table 11:** Tissues distribution of canine distemper virus antigens in thyroid, parathyroid, pancreas, adrenal, and heart of free-ranging black-tufted marmosets (*Callithrix penicillata*) naturally infected with the canine distemper virus.

Other organs		ID							Total
		1	2	3	4	5	6	7	
Thyroid	Follicular cells	+	+	+	++	+	+	NE	6/6
	Colloid	+	+	+	++	+	+	NE	6/6

Parathyroid	Chief cells	NP	NP	+++	NP	+++	+++	NE	3/3

Pancreas	Exocrine cells (epithelial cells)	++	NE	NE	NE	++	NE	NE	2/2
	Endocrine cells (Islets of Langerhans)	0	NE	NE	NE	+	NE	NE	1/2
	Ducts	+	NE	NE	NE	+	NE	NE	2/2
	Connective tissue	+	NE	NE	NE	+	NE	NE	2/2

Adrenal	Cortical cells	++	+	++	+	+	0	+	6/7
	Medullary cells	++	+	+	0	+	0	0	4/7
	Endothelial cells	+	+	+	0	0	0	0	3/7

Heart	Endocardium	+	0	+	+	+	+	NE	5/6
	Myocardium	+	+	+	+	+	+	NE	6/6
	Epicardium	+	+	+	+	+	+	NE	6/6
	Endothelial cells	+	0	0	0	0	0	NE	1/6

*Note:* 0: negative; +: mild, focal or multifocal; ++: moderate, focal or multifocal; +++: intense multifocal to coalescent or diffuse.

Abbreviations: NE, not evaluated; NP, not present.

## Data Availability

The data that support the findings of this study are available from the corresponding author upon reasonable request.

## Supporting Information

Supporting Information 1 Video S1: Generalized myoclonus observed in a black-tufted marmoset (Callithrix penicillata) naturally infected with canine distemper virus (CDV), during an outbreak in a forested urban area at the Belo Horizonte Zoo, Brazil, in 2022.

Supporting Information 2 Figure S1: Anti-canine distemper virus (CDV) immunohistochemistry positive (A) and negative (B–F) controls. (A) Giant anteater (*Myrmecophaga tridactyla*) lung with immunostained bronchiole epithelial cells, pneumocytes and alveolar machrophages. Magenta chromogen, 200×. Insert: negative control of the same animal. Magenta chromogen, 400×. (B–F) Negative controls of CDV-positive free-ranging black-tufted marmosets (*Callithirx penicillata*). (B) Negative control of CDV-positive brain. Magenta chromogen, 400×. (C) Negative control of CDV-positive skin. Magenta chromogen, 200×. (D) Negative controls of CDV-positive tongue. Magenta chromogen, 400×. (E) Negative control of CDV-positive spleen. Magenta chromogen, 100×. (F) Negative controls of CDV-positive urinary bladder. Magenta chromogen, 100×.

Supporting Information 3 Figure S2: Anti-herpesvirus immunohistochemistry from free-ranging black-tufted marmosets (*Callithrix penicillata*) naturally infected with canine distemper virus. (A) Brain with multiple cells with nuclear immunostained cells. Magenta chromogen, 400×. (B) Tongue with multifocal immunostained epithelial cells. Magenta chromogen, 400×.
