# Supplementary figures and images for: Lethal Canine Distemper Virus (Morbillivirus canis) Outbreak in Free-Ranging Black-Tufted Marmosets (Callithrix penicillata) in Brazil: Clinical, Pathological, Genotypical Evaluation, and Assessment of Viral Tropism
Source: Transbound Emerg Dis. 2025 Nov 12;2025:4701926. doi: 10.1155/tbed/4701926 (PMC12629703; doi:10.1155/tbed/4701926)

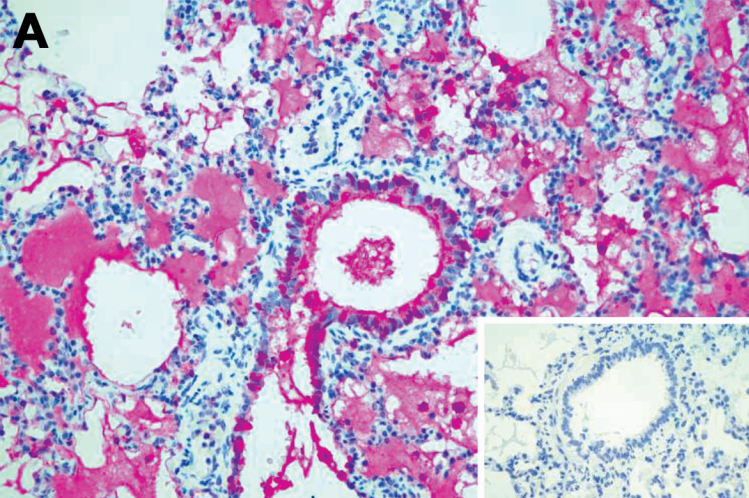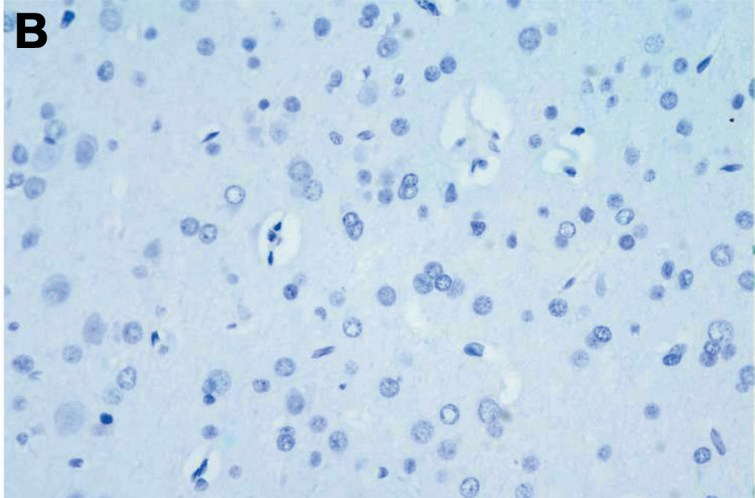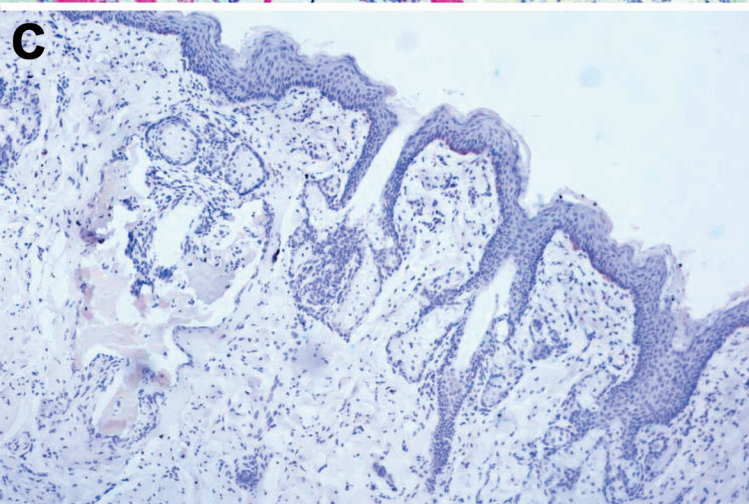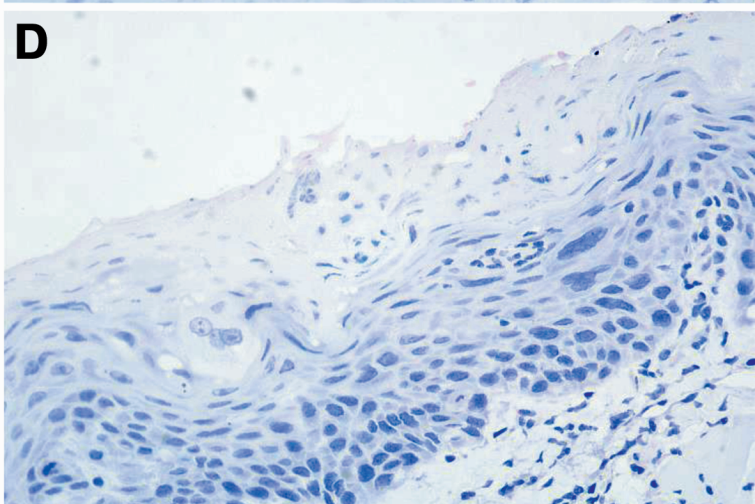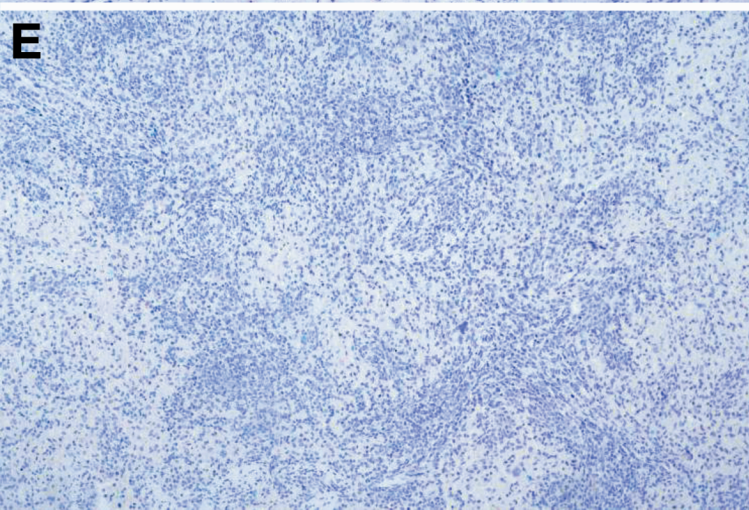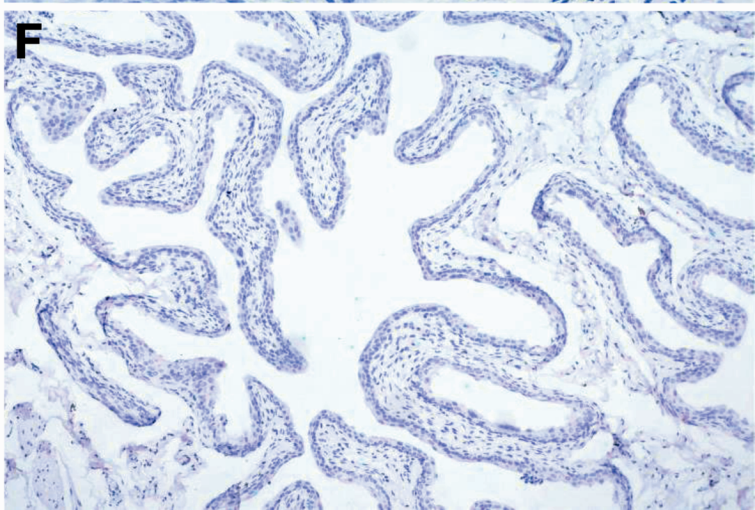

Supplement: Supporting Information 2 — Figure S1: Anti-canine distemper virus (CDV) immunohistochemistry positive (A) and negative (B–F) controls. (A) Giant anteater (Myrmecophaga tridactyla) lung with immunostained bronchiole epithelial cells, pneumocytes and alveolar machrophages. Magenta chromogen, 200×. Insert: negative control of the same animal. Magenta chromogen, 400×. (B–F) Negative controls of CDV-positive free-ranging black-tufted marmosets (Callithirx penicillata). (B) Negative control of CDV-positive brain. Magenta chromogen, 400×. (C) Negative control of CDV-positive skin. Magenta chromogen, 200×. (D) Negative controls of CDV-positive tongue. Magenta chromogen, 400×. (E) Negative control of CDV-positive spleen. Magenta chromogen, 100×. (F) Negative controls of CDV-positive urinary bladder. Magenta chromogen, 100×. [file 4701926.f2.pdf]

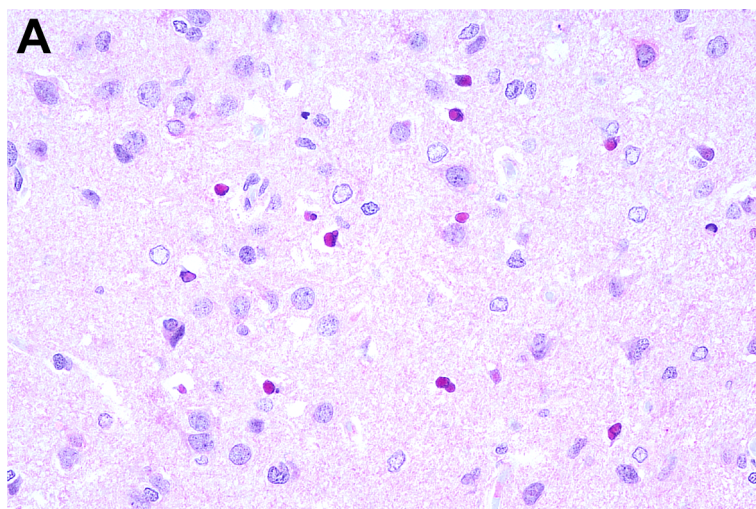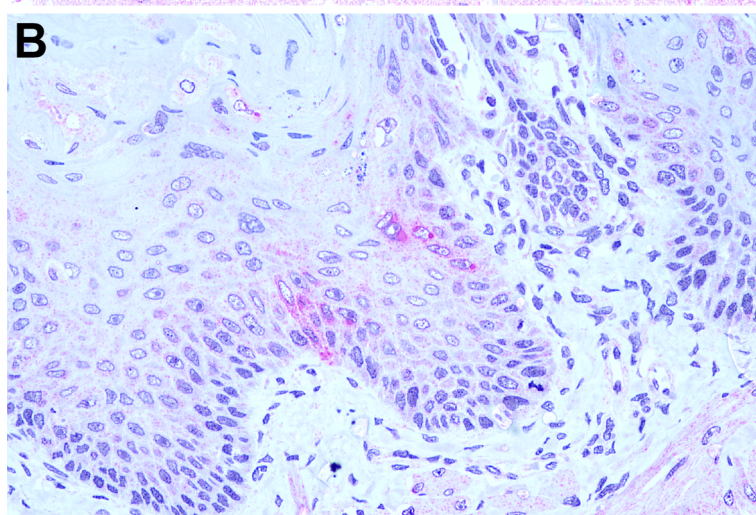

Supplement: Supporting Information 3 — Figure S2: Anti-herpesvirus immunohistochemistry from free-ranging black-tufted marmosets (Callithrix penicillata) naturally infected with canine distemper virus. (A) Brain with multiple cells with nuclear immunostained cells. Magenta chromogen, 400×. (B) Tongue with multifocal immunostained epithelial cells. Magenta chromogen, 400×. [file 4701926.f3.pdf]
